# Genomic insights into the probiotic potential of dairy-associated *Saccharomyces cerevisiae* WUT3 and WUT151 strains

**DOI:** 10.1007/s10142-026-01963-4

**Published:** 2026-07-06

**Authors:** Aleksander Gryciuk, Małgorzata Milner-Krawczyk, Adrianna Skoneczna, Jolanta Mierzejewska

**Affiliations:** 1 Laboratory of Microbiology and Bioengineering, Faculty of Chemistry, Warsaw University of Technology, Warsaw, Poland; 2 Institute of Biochemistry and Biophysics, Polish Academy of Sciences, Warsaw, Poland

**Keywords:** *Saccharomyces cerevisiae*, *Saccharomyces boulardii*, Next-generation sequencing, Comparative genomics, Probiotics, Genomic variation

## Abstract

Despite the prevalence of bacterial probiotics, yeast-based preparations offer unique therapeutic benefits that remain a key research priority. Here, we conducted a comparative genomic characterization of the dairy-associated *Saccharomyces cerevisiae* WUT3 and WUT151 strains, building on previous evidence showing their enhanced probiotic potential relative to the probiotic *S. cerevisiae* var. *boulardii* CNCM I-745. The genomes of WUT3 and WUT151 were compared with the reference *S. cerevisiae* S288C and the probiotic *S. boulardii* CNCM I-745. At the CDS level, both WUT strains were closer to S288C, suggesting that their probiotic properties stem from unique mechanisms rather than general genetic similarities to the probiotic strain. WUTs shared selected probiotic-related features, including *ENA1* deletions, *WHI2* alterations, and loss of Ty1/Ty2 regions present in S288C. Together with conserved core stress-response genes, this profile supports their tolerance to low pH, bile salts, and elevated temperature. Gene content analysis revealed redundancy within the hexose transporters and confirmed the absence of the *ASP3* cluster. Indels in *HSP150* and *PIR3* may reflect WUT and *S. boulardii* cell wall variations. Overall, the probiotic potential of WUT strains does not originate from a single ‘probiotic genome’, but rather from a unique combination of traits dispersed throughout the *S. cerevisiae* species. Since no detrimental features were revealed in the analyzed genomes, these strains represent promising candidates for further clinical evaluation. Furthermore, these findings demonstrate that integrative genomic analysis is an effective strategy for the identification, selection, and characterization of candidate probiotic yeast strains.

**Supplementary Information:**

The online version contains supplementary material available at 10.1007/s10142-026-01963-4.

## Introduction

Fermented milk and other dairy products constitute niches rich in microorganisms with functional and technological relevance (Tamang et al. 2020; Tamang and Lama 2022). Although probiotic research has historically focused on lactic acid bacteria, recent studies indicate that yeasts, including *Saccharomyces cerevisiae*, may also exhibit probiotic and other health-related traits. This supports the exploration of food-associated yeast isolates as candidate probiotic strains for further functional and genomic characterization (Lama and Tamang 2022; Gryciuk et al. 2026; Lama et al. 2026).

*Saccharomyces cerevisiae* var. *boulardii* CNCM I-745 is the only yeast strain officially approved as a probiotic. It can survive a wide range of pH and temperature conditions and has been documented to have beneficial effects on human health (Gopalan et al. 2023). It was debated whether SB should be considered a separate species. Some reports pointed out differences in phenotype, genomics, and functional characteristics, suggesting that SB represents a species distinct from *Saccharomyces cerevisiae* (Duffey et al. 2025). However, as early as 2003, one of the first comparative studies demonstrated that the D1/D2 domain of 26S rDNA, the ITS1-5.8S rDNA-ITS2 region, and other commonly used phylogenetic markers were over 99.9% identical between *S. boulardii* and *S. cerevisiae* (Van Der Aa Kühle and Jespersen 2003). More recently, whole-genome comparisons based on Average Nucleotide Identity (ANI) have further supported the classification of *S. boulardii* as a subspecies of *S. cerevisiae* (Pais et al. 2021). Additionally, large-scale population genomic analyses place *S. boulardii* within the Wine-European lineage of *S. cerevisiae* rather than as a separate species-level lineage (Peter et al. 2018). Due to this close relationship, the taxonomic debate is largely over, and it is officially recognized as a variant (or strain) within the *S. cerevisiae* species. Nevertheless, other *Saccharomyces* sp. strains remain largely unexplored with respect to their probiotic properties (Kunyeit et al. 2020).

Yeast probiotic properties are correlated with multiple factors, such as enhanced thermotolerance, acid and osmotic resistance, improved adhesion, and antimicrobial properties (Pais et al. 2021; Ting et al. 2026). The genetic basis of *S. boulardii* probiotic features remains unclear, whereas some reports have shown the genomic variations that may be advantageous for *S. boulardii*. A recent study correlated acid tolerance and antimicrobial activity with acetic acid production in *S. boulardii*. The variants in *SDH1* (F317Y) and *WHI2* (Ser287*) decreased acid tolerance but increased antimicrobial activity (De Carvalho et al. 2024). Another report focused on yeast adhesion, highlighting the flocculation gene family, with *FLO1*, *FLO5*, and *FLO10* increasing cell-to-cell adhesion, and *FLO11* affecting cell-to-surface adhesion (Mohammadi and Saris 2023). The impact of genetic variations in stress-response genes is not straightforward. For instance, deletion of the *ENA1* gene in *S. boulardii* reduced tolerance to high salt concentrations. However, the strain still survived gastric fluid, while its virulence in the in vivo model was reduced, resulting in an overall increase of probiotic potential (Imre et al. 2026). Thermotolerance of *S. boulardii* is defined by multiple factors. It was shown that *S. boulardii* encodes the most important heat shock proteins, often with higher copy numbers than in *S. cerevisiae*. It was also shown that a nonsense mutation in the *PGM2* gene conferred higher thermotolerance in *S. boulardii*, while limiting its galactose utilization (Ting et al. 2026). A notable research gap exists regarding the whole-genome analyses of probiotic yeast. Comparative genomics between *S. cerevisiae* and *S. boulardii* is limited by incomplete functional annotation databases (Duffey et al. 2025).

Our previous work demonstrated that two dairy-associated *S. cerevisiae* strains, WUT3 and WUT151 (hereafter referred to as WUT strains), displayed probiotic properties comparable to or exceeding those of *S. boulardii*. WUT and *S. boulardii* showed similarities in gastrointestinal tract survival rates, antioxidation activity, and anti-pathogen activity, without toxicity to human cells in vitro (Gryciuk et al. 2026). Both WUT strains displayed enhanced thermostability, growing at 37 °C. In this study, we employed comparative genomics to characterize the nuclear and mitochondrial genomes of WUT3 and WUT151. The comparison with *S. cerevisiae* S288C provided a standardized reference framework for identifying sequence and structural variation within the species. Comparison with the probiotic strain *S. cerevisiae* var. *boulardii* CNCM I-745, in turn, allowed us to evaluate whether WUT3 and WUT151 share genomic features associated with probiotic potential.

## Materials and methods

**Fig. 1:**
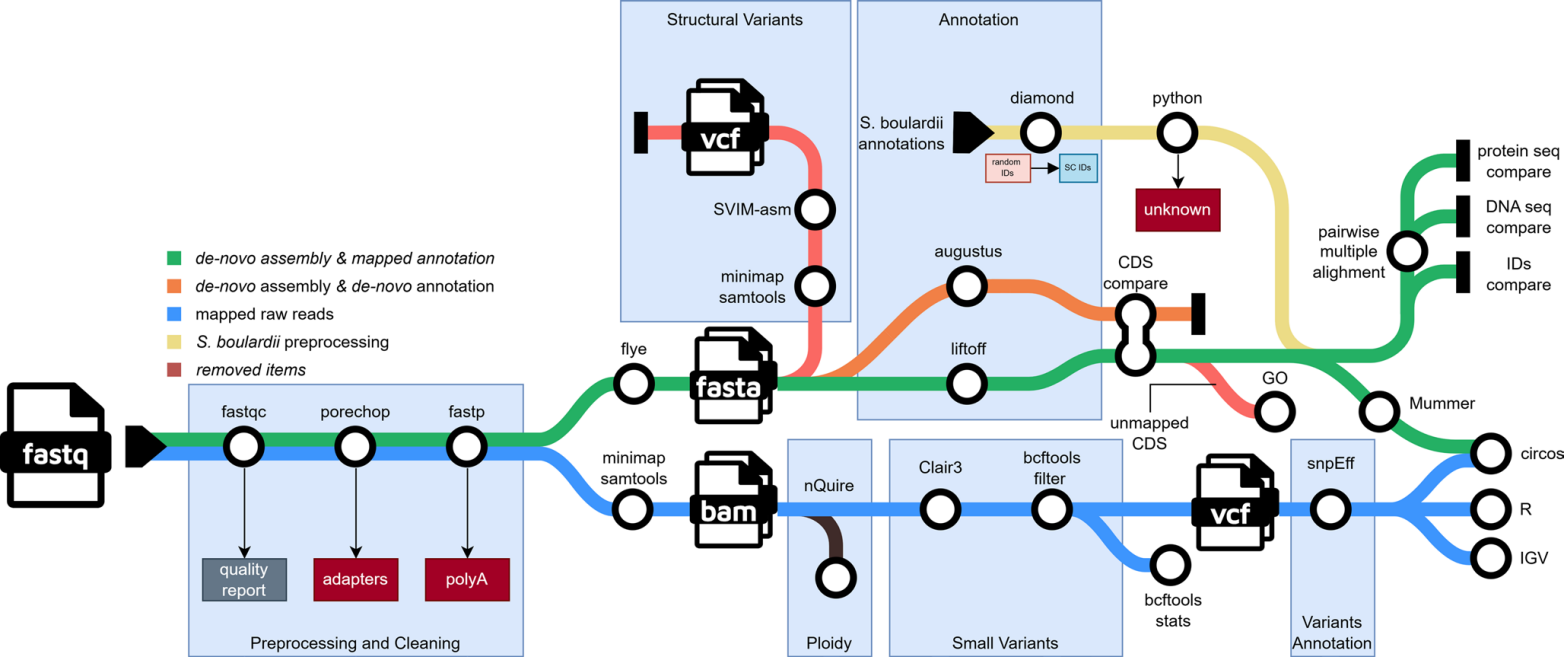
Schematic overview of the analytical workflow used in this study. The workflow included read preprocessing, genome assembly, read mapping, ploidy estimation, small variant calling, assembly-based structural variant detection, annotation transfer, de novo gene prediction, and comparative CDS analysis. The figure was prepared using a graphical template designed by Fellows Yates JA, Garcia M, and Le Nézet L for nf-core

A schematic overview of the analytical workflow used in this study is presented in Fig. 1. The analysis combined read-based variant calling, assembly-based structural variant detection, annotation transfer, *de novo* gene prediction, and comparative CDS analysis.

### Yeast strains

The genome analysis was conducted on WUT3 and WUT151 *Saccharomyces cerevisiae* strains, provided from the Warsaw University of Technology Yeast Collection (https://wutyeastcollection.pw.edu.pl/) (Gryciuk et al. 2026). WUT3 was isolated from kefyr in Turkey, and WUT151 was isolated from mare’s milk in Kyrgyzstan. For the laboratory examination (PCR of *HXT* genes), the BY4742 strain derived from the *S. cerevisiae* S288C strain (*MATα his3Δ1 leu2Δ0 lys2Δ0 ura3Δ0*), and *S. boulardii* CNCM I-745 were used as the reference. For flow cytometry, the strains WUT3 ρ0 and WUT151 ρ0 were prepared by low ethidium bromide exposure - three passages in liquid YPD (1% w/v yeast extract, 2% w/v peptone, 2% w/v glucose) supplemented with 0.4 µg/ml ethidium bromide. Strains that were not growing on YPG medium (1% yeast extract, 2% peptone, 2% glycerol) and had no other than a nuclear signal after propidium iodide staining in fluorescence microscopy analysis were qualified as ρ0. BY4741 (*MATa his3Δ1 leu2Δ0 met15Δ0 ura3Δ0)* and BY4743 (*MATa/MATα his3Δ1/his3Δ1 leu2Δ0/leu2Δ0 lys2Δ0/LYS2 met15Δ0/MET15 ura3Δ0/ura3Δ0*) and BY4743 ρ0 (Skoneczna et al. 2007) served as flow cytometry controls.

### Reference genomes

The *Saccharomyces cerevisiae* S288C genome comprised the nuclear genome assembly (NCBI GCF_000146045.2) supplemented with the mitochondrial genome (NCBI NC_001224.1), together with corresponding annotation files. Hereafter, this dataset is referred to as the reference genome, unless stated otherwise.

The *Saccharomyces cerevisiae* var. *boulardii* CNCM I-745 genome comprised the nuclear genome assembly (NCBI ASM129837v2) with the corresponding annotation file. Hereafter, this dataset is referred to as the probiotic reference, unless stated otherwise.

### Genome sequencing

Genomic DNA was sequenced using Oxford Nanopore Technology by an Oxford Nanopore Certified Service (Genexon, Poland), using the GridION platform. Data acquisition was performed with MinKNOW v24.06.10 (MinKNOW Core v6.0.8; Bream v8.0.9; Configuration v6.0.13), and basecalling was performed with Dorado v7.4.12 using the sup@v4.3.0 model. De novo genome assembly was performed using Flye (Kolmogorov et al. 2020) and Unicycler (Wick et al. 2017a), followed by polishing with Medaka. Assembly completeness was evaluated using BUSCO (v5.7.1) (Tegenfeldt et al. 2025) with the saccharomycetes_odb10 database, running in euk_genome_min mode, using miniprot as the gene predictor. As a result, raw reads files (fastq) and *de novo* assemblies’ files (fasta) were obtained for each strain. The fastq files are available on the ENA database (PRJEB102465).

### Raw reads cleaning

Raw read quality was assessed using FastQC (v0.12.1) (Andrews 2010) and MultiQC (v1.17) (Ewels et al. 2016). Reads were trimmed using Porechop (v0.2.4) (Wick et al. 2017b) with default settings, excluding reads with adapters in the middle (--discard_middle). As QC reports indicated the presence of poly(X) tails, trimmed reads were further processed using FastP (v0.22.0) (Chen 2025), with poly(X) trimming enabled (--trim_poly_x, --poly_x_min_len 20), tail trimming (--cut_tail), a minimum read length of 1000 bp (--length_required 1000), cut window of 50 (--cut_window_size 50), and a cut mean quality of 9 (--cut_mean_quality 9). Default FastP adapter trimming was disabled (-A) because adapter removal had already been performed by Porechop. The QC after cleaning showed a minor improvement.

### Read mapping to the reference genome

Cleaned reads were mapped to the *S. cerevisiae* S288C reference genome. The reference was indexed using minimap2 (v2.28) (Li 2018). Reads were aligned using minimap2 with the ONT preset (-ax map-ont), followed by sorting and indexing the BAM files using Samtools (v1.6) (Danecek et al. 2021).

### Ploidy verification

In silico examination of WUT3 and WUT151 ploidy was performed using nQuire (Weiß et al. 2018). This was considered an exploratory approach, as nQuire was originally designed for short-read NGS, rather than ONT. Therefore, flow cytometry was performed to estimate DNA content profiles.

The DNA content of yeast cells was measured similarly to (Krol et al. 2018). Briefly, the 1 ml aliquots of a yeast exponential culture (5 × 10^6^ −1 × 10^7^ cells/ml) were spun down and fixed in 1 ml of chilled (−20° C) 80% ethanol (Polmos, Warsaw, Poland) for 2 h. The fixed cells were washed twice in FACS buffer (0.2 M Tris-HCl (Sigma-Aldrich), pH 7.4, 20 mM EDTA (Merck, Darmstadt, Germany) and incubated for 2 h at 37 °C in FACS buffer with 1 mg/ml RNase A (Sigma-Aldrich) to eliminate RNA from samples. Then the cells were washed with phosphate-buffered saline (PBS) and stained for 1 h at 4 °C in the dark with 100 µl of propidium iodide solution (10 µg/ml, in PBS; Calbiochem). After staining, 900 µl of PBS was added, and the cells were sonicated (3 × 10 s) in an ultrasonic bath (Branson). FACS analysis of DNA content was performed using a FACSCalibur analyzer (Becton, Dickinson and Company, Franklin Lakes, NJ, USA). A total of 10,000 cells were counted in a single assay. At least three biological repetitions were made for each sample.

### Variant identification and annotation

Variants were identified using Clair3 (v1.0.10) (Zheng et al. 2022) on the Galaxy Europe platform (usegalaxy.eu), with the standard settings for ONT data (--platform=’ont’, model=r1041_e82_400bps_sup_v500) using the S288C reference genome (including mitochondrial sequence). Then, variants were filtered using bcftools (v1.15.1) (Danecek et al. 2021) with the following criteria: DP > = 10, GQ > = 20.

For the variant annotation, the SnpEff (v5.4) (Cingolani et al. 2012b) database was built using the reference S288C genome and GFF annotation file. Subsequently, variants were annotated using the SnpEff ann function, with upstream and downstream set to 200 bp (-ud 200). For the downstream processing, a tabular summary was created using SnpSift (v5.4) (Cingolani et al. 2012a).

### Variant downstream analysis and visualization

Variant calling results were analyzed in R (v4.5.2) using the tidyverse (v2.0.0) (Wickham et al. 2019) and ComplexUpset (v1.3.3) (Michał Krassowski et al. 2022) packages. The variants’ hotspots were identified for high- and moderate-impact variant regions within 5-kb windows. The window was considered a hotspot if the SNP density exceeded the 99th percentile of the global variant density distribution.

### Genome structural comparison

The generic structural comparison metrics for assembled WUT genomes and the S288C reference genome were obtained with MUMmer dnadiff (v4.0.1) (Marçais et al. 2018) using default settings. Structural variants (SVs) were identified using the SVIM-asm (v1.0.3) (Heller and Vingron 2021). First, assembled genomes were mapped to the reference S288C genome using minimap2 with the recommended settings from the SVIM-asm developers: -a, -x asm5, --cs, -r2k, creating BAM and PAF files. Then, variants were called using the BAM file and SVIM-asm with default settings. Variants without the SV type were then filtered out. Downstream processing was performed using R with the tidyverse package. The PAF file was used to assess genome collinearity.

### Targeted analysis of probiotic-associated variants

Following genome-wide SNP and structural variant detection, a targeted analysis was performed. Genes of interest were selected based on published reports linking them to probiotic-relevant features: thermotolerance, antimicrobial activity, resistance to osmotic stress, and adhesion. For each selected gene, moderate- and high-impact SNPs/indels and structural variants were reviewed using IGV (Robinson et al. 2023) to confirm their genomic context.

### Genome structure visualization

The genome visualization was created using Circos (v0.69-10) (Krzywinski et al. 2009). Interchromosome alignments were identified by filtering the PAF file (from genome mapping) for records with different query and template chromosome names, lengths greater than 5 kb, and mapping quality greater than 20. Insertions and deletions were marked based on the SVs data from the VCF file.

Additional genome visualization (supplementary materials) was performed to present the annotated genome features. The mDNA, tDNA, rDNA, CDS, and pseudogene densities were calculated in 20 kb windows from reference-based GFF annotations (described below) using bedtools (v2.18) (Quinlan and Hall 2010). GC content (from bedtools nuc) and SNP density (from dnadiff) were calculated in 5 kb windows. The results were then visualized in Circos.

### Reference-based genome annotation

WUT3 and WUT151 genome assemblies were annotated using liftoff (v1.6.3) (Shumate and Salzberg 2021) with the *S. cerevisiae* S288C genome and annotation. Multiple copies of the features were allowed (-copies). Annotation summary metrics (mapped and unmapped features) were obtained from the liftoff report. Feature counts (CDS statistics) were computed from the corresponding GFF annotation files, using standard Unix command-line utilities.

### *De novo* genome annotation

WUT assemblies were annotated *de novo* using AUGUSTUS (v3.2.3) (Stanke et al. 2008). *Saccharomyces cerevisiae* S288C model was used (--species=saccharomyces_cerevisiae_S288C) with the default transition matrix.

### Identification of novel CDS

We used a custom pipeline to identify novel CDS in WUT assemblies by comparing the *de novo* predictions to reference-based annotations. CDS sequences were extracted from Liftoff and AUGUSTUS GFF files, using AGAT *agat_sp_extract_sequences.pl* (v0.8.1) (Jacques Dainat et al. 2026), restricted to CDS features (--type CDS). Sequences were converted to the MMseqs2 (Kallenborn et al. 2025) database using mmseqs createdb and compared using MMseqs2 search (v15) in nucleotide mode (--search-type 4). CDS were classified as novel when present in the AUGUSTUS set but absent in liftoff one. Novel CDS were translated into proteins using EMBOSS transeq (v6.6.0.0) (Rice et al. 2000), and annotated using NCBI BLASTp (web service) (Camacho et al. 2009), with default settings to identify known homologs and functional annotations.

### Gene ontology enrichment and functional analysis

The GO analysis was performed on genes not mapped by liftoff, using ShinyGO (v0.82, organism: *S. cerevisiae*) (Ge et al. 2020) and g: Profiler (v113, organism: *S. cerevisiae*, database updated on 23.05.2025) (Kolberg et al. 2023). Enrichment was computed for GO Biological Process, Molecular Function, Cellular Component, and for KEGG pathways. Terms were considered significantly at FDR (ShinyGo) or padj (g: Profiler) below 0.05. Additionally, g:Profiler results were filtered to retain terms with *p* < 10^− 4^ in at least one strain. For visualization, the top 10 terms per strain (lowest FDR) were selected and plotted using OriginPro 2021 (v9.8.5.212).

### PCR verification

PCR verification was performed to confirm the absence of the *ASP3-4* gene and selected representative *HXT* genes (*HXT1*, *HXT6*, *HXT8*). Genomic DNA from WUT3, WUT151, and BY4742 (derivative of S288C) was extracted using the Bacterial & Yeast Genomic DNA Purification Kit (EURx, E3580). Isolation efficiency and DNA quality were evaluated by agarose gel electrophoresis and NanoDrop (Thermo Fisher Scientific). Primers were designed using the NCBI Primer-BLAST and are listed in Table 1. Primer specificity was assessed in silico using FastPCR (v6.9.0.22). PCR was performed using DreamTaq DNA Polymerase (Thermo Fisher Scientific, EP0701), according to the manufacturer’s protocol. The thermocycler program was set as follows: 95 °C (2 min), [95 °C (30 s), 53 °C (1 min), 72 °C (1 min)] × 36, 72 °C (7 min), 4 °C. The results were visualized using gel electrophoresis (2% (w/v) agarose in 1x TAE buffer, 100 V, 30 min).

**Table 1:** Primers used for PCR

Gene	Forward	Reverse
*ASP3-4*	ACGGTATCTCCGAGGCACTA	GTAGCCAGATCCAATGGCGT
*HXT1*	CGTTTTGGCCGTCGTAACTG	TAGTCAGCGCCTCTCTTGGA
*HXT6*	AAGAGCACGAACCTGTCGTT	AGCCCAGGCAAAACACAAAC
*HXT8*	TTTCGGAAACTGCGCCAAAG	CATGGTGGCTGCTCCAAGTA

The presence of the HXT8 CDS in the WUT3 genome was additionally verified using local BLAST+ (v2.16.0) (Camacho et al. 2009). The WUT3 genome database was created using the makeblastdb tool. Then, two BLAST runs were conducted, first with *the HXT8 sequence and second with the HXT7* sequence as a query.

### ID mapping and preprocessing

The comparative analysis with the probiotic strain was performed using the S288C reference genome and probiotic reference (see Reference genomes), together with WUT3 and WUT151 assemblies. Genes were linked to their corresponding standard IDs using SGD. For SB, gene identifiers were first linked to S288C IDs using DIAMOND (v2.1.13) (Buchfink et al. 2021). Entries lacking all: name, gene, and protein identifiers (or flagged as “unknown” in the protein section) were excluded.

### Comparison of gene content

The first analysis compared gene presence across WUT strains, genome reference, and the probiotic reference, based on shared gene IDs (see Reference genomes). We compiled the union of genes present in at least one genome and generated a binary membership matrix indicating the presence or absence in each genome. Relationships between strains were visualized using an UpSet plot (v0.9.0, Python v3.11.12) (Lex et al. 2014). Jaccard index was used to quantify pairwise similarity in gene content:

$$\:J({A}_{1},\:\dots\:,\:{A}_{n})=\frac{\left|{\bigcap\:}_{i=1}^{n}{A}_{i}\right|}{\left|{\bigcup\:}_{i=1}^{n}{A}_{i}\right|}$$

Where: J – Jaccard index; A_i_ – gene set of strain i.

### CDS sequence comparison

Analysis was based on shared CDS sequences of WUT strains, the reference strain, and the probiotic reference (see Reference genomes). Pairwise global alignments were computed separately for nucleotide and amino acid sequences, using biopython’s PairwiseAligner (v1.85) (Cock et al. 2009). Nucleotide alignments scoring was set as follows: match = 1, mismatch = −1, gap open = −10, gap extend = −0.5. For protein alignment, BLOSUM62 was used with the same gap penalties. Identity (ID) represents the proportion of identical residues to the total sequence length. Similarity (SIM) was defined as the alignment score divided by the maximum possible score (normalized alignment score):

$$\:\mathrm{S}\mathrm{I}\mathrm{M}=\frac{S\left(se{q}_{1},\:se{q}_{2}\right)}{\mathrm{max}\left(S\left(se{q}_{1},\:se{q}_{1}\right),\:S\left(se{q}_{2},se{q}_{2}\right)\right)}\:$$

Where: S – the alignment score for the given sequences.

Genes with low similarity (SIM < 0.8) were selected for functional analysis. With the use of the Saccharomyces Genome Database Gene Association File (GAF v2.2, GO release 2025-03-16, PANTHER v19.0), genes were mapped to GO terms and namespaces (aspects). GO term depth (hierarchical level) was assigned using the Gene Ontology OBO file (v1.2, release 2025-07-22). To reduce redundancy from general GO terms, analysis was limited to intermediate term depth (levels 3–6). GO term occurrences were counted per comparison, normalized by the number of mapped GO annotations, and reported as mean ± SEM across comparisons. The data visualization was conducted using seaborn (v0.13.2) (Waskom 2021) and matplotlib (v3.10.3) (Hunter 2007) in Python. Strongly diverged proteins were highlighted using the criterion requiring SIM < 0.5 in all WUT comparisons. For genes, the criterion allowed one WUT comparison to exceed the 0.5 threshold.

## Results

### Raw reads analysis and variant calling

To evaluate genetic diversity between the studied WUT strains and the reference *S. cerevisiae* S288C, a variant-calling analysis was performed on raw Nanopore reads. After quality filtering, the reads were mapped to the reference genome to identify single-nucleotide polymorphisms (SNPs) and small structural differences. The sequencing data for both strains passed the quality control requirements (Table 2). The lower read number, total bases, and raw-read GC content of WUT3 mainly reflected technical differences in sequencing yield and coverage depth rather than biological differences between the strains. The median read length was characteristic of Nanopore sequencing and exceeded 7 kb, with a median quality of over Q37. Raw fastq files are available on the European Nucleotide Archive (ENA) database (PRJEB102465; www.ebi.ac.uk). The reference genome of *S. cerevisiae* S288C (R64, GCF_000146045.2) was used for comparative analyses. Prior to the variant calling, the ploidy of strains was estimated using nQuire. WUT151 was identified as diploid (r^2^ > 0.96), while results for WUT3 were mixed. To further investigate this, we performed a flow cytometry analysis. Both strains displayed similar DNA content profiles, which were slightly shifted relative to the 2n reference (Fig. S1). The observed shift may result from increased mitochondrial DNA content or may indicate a predominantly diploid state with atypical DNA content.

**Table 2:** Quality control results of WUT3 and WUT151 whole genome sequencing

Parameter	WUT3	WUT151
Total sequences	46 551	68 325
Total bases	573.8 Mbp	> 1 Gbp
Raw-read GC content (%)	33	36
Median length (kb)	7	12
Median quality (Q)	37	38
Duplicates	2.1%	0.7%

To nail this issue, we prepared ρ0 derivatives of the WUT3 and WUT151 strains lacking mitochondrial DNA (mtDNA) using the low-ethidium bromide exposure method. Subsequent flow cytometry analysis of propidium iodide-stained cells showed a clear shift in the DNA content histograms to the left, to a position similar to that of BY4743 ρ0; thus, the initially observed DNA content enrichment in WUT3 and WUT151 may indeed result from increased mtDNA content in these strains (Fig. S1). This explanation seems correct for the WUT151 strain, as WUT151 ρ0 and BY4743 ρ0 DNA content histograms nearly overlap. In contrast, the WUT3 ρ0 DNA content histogram remains slightly shifted to the right compared to the BY4743 ρ0 control, suggesting a higher genomic DNA content in this strain. This difference in DNA content can be explained by an additional DNA segment in its genome that is absent in the reference strain, or by aneuploidy. And indeed, some extra DNA sequences are present in the WUT3 genome, as it is 110 kbp longer than the WUT151 genome. Interestingly, the tendency for losing mtDNA in both strains is different; when WUT151 is producing ρ0 cells quite easily (about ~ 37% of cells lost the mtDNA during three passages on low ethidium bromide), WUT3 keeps its mtDNA more tightly (only ~ 8% of cells lost the mtDNA in the same conditions) (Fig. S1).

Thus, WUT3 and WUT151 were treated as diploids in the subsequent variant analysis.

Mapping efficiency achieved 96.3% for WUT3 and 99.3% for WUT151, with mean coverage depths of 44× and 87×, respectively. WUT3 showed a lower variant rate (1 variant per 170 bp) than WUT151 (1 variant per 126 bp). SNPs constituted the majority of variants, with 65,585 detected in WUT3 and 88,598 in WUT151. Indels accounted for a smaller fraction of 5,518 and 7,500 variants, respectively. Variant annotation revealed that over 75% of all variants had low impact or addressed the gene regulatory regions (Table 3).

**Table 3:** Variants impact based on SnpEff annotation

Impact	WUT3	WUT151
High	621 (0.87%)	832 (0.87%)
Moderate	16179 (22.8%)	23021 (24.0%)
Low	23963 (33.8%)	31777 (33.2%)
Modifier	30224 (42.6%)	40149 (41.9%)

**Fig. 2:**
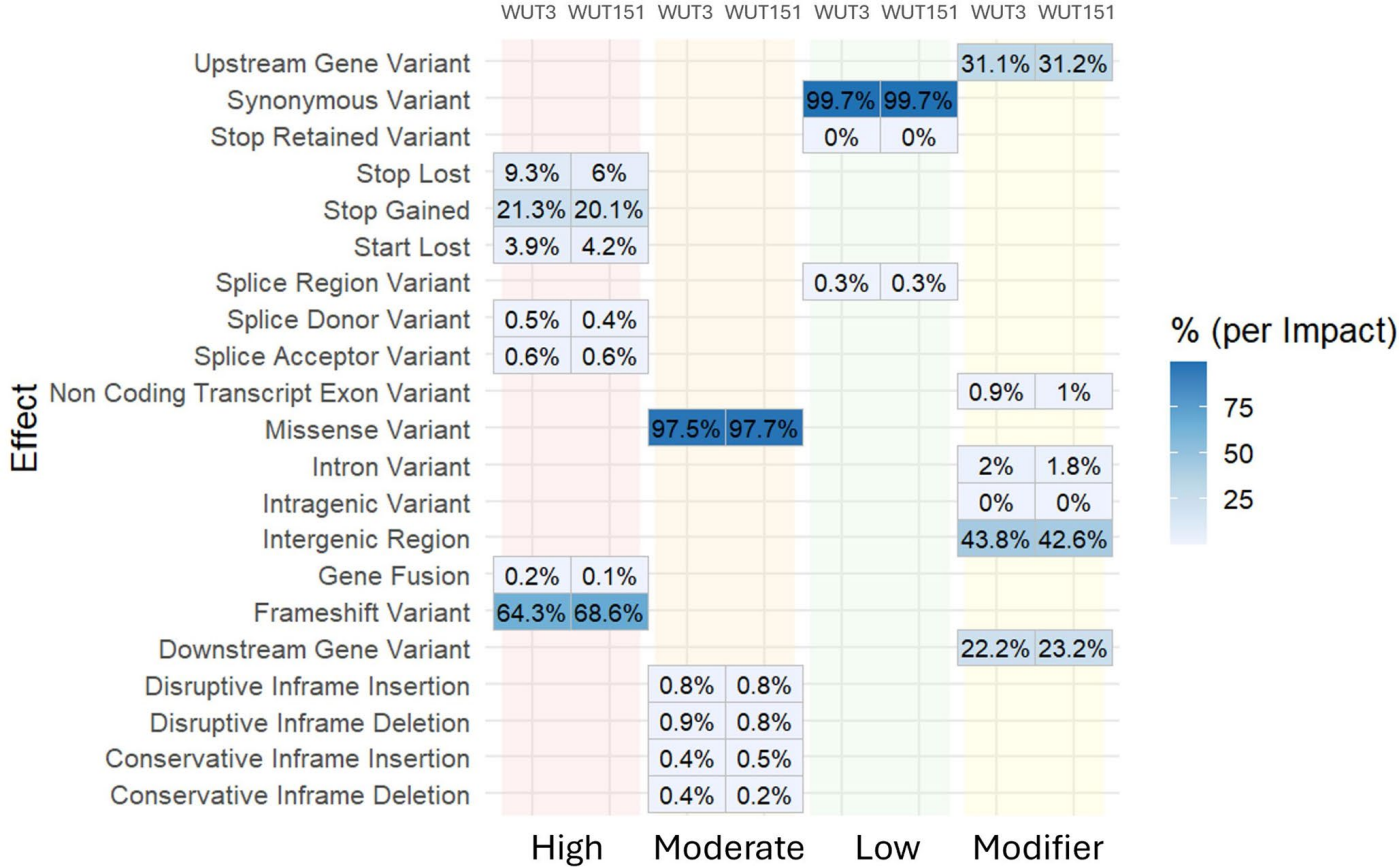
Distribution of functional effects across variant impact categories. Percentages indicate the relative contribution of each effect within the respective impact group

High-impact variants were predominantly associated with frameshift and nonsense mutations (Fig. 2). Almost all moderate-impact variants were classified as missense mutations, with only minor fractions attributed to indels. Most low-impact variants were identified as synonymous mutations, whereas modifier variants were in upstream, downstream, or intergenic regions.

WUT151 contained more variants per 1 Mbp than WUT3 in each chromosome (6,112, 8,171, respectively), except for the mitochondrial sequence (13,768, 12,043, respectively), with the greatest difference at chromosome III, and the least at chromosome X. Among the most affected genes (containing at least one high-impact variant), 122 were unique to WUT3, 243 were unique to WUT151, and 238 were shared (Fig. S2). The genes *GAL1*, *BI2*, *SRL2*, *AI4*, *FRE2*, and *EMP46* were the most disrupted, containing the most high-impact variants. It was also notable that genes associated with transposons (e.g., *YER138C*), pseudogenes (e.g., *YFL056C*), or genes of unknown function (e.g., *YHL008C*) were highly changed.

**Fig. 3:**
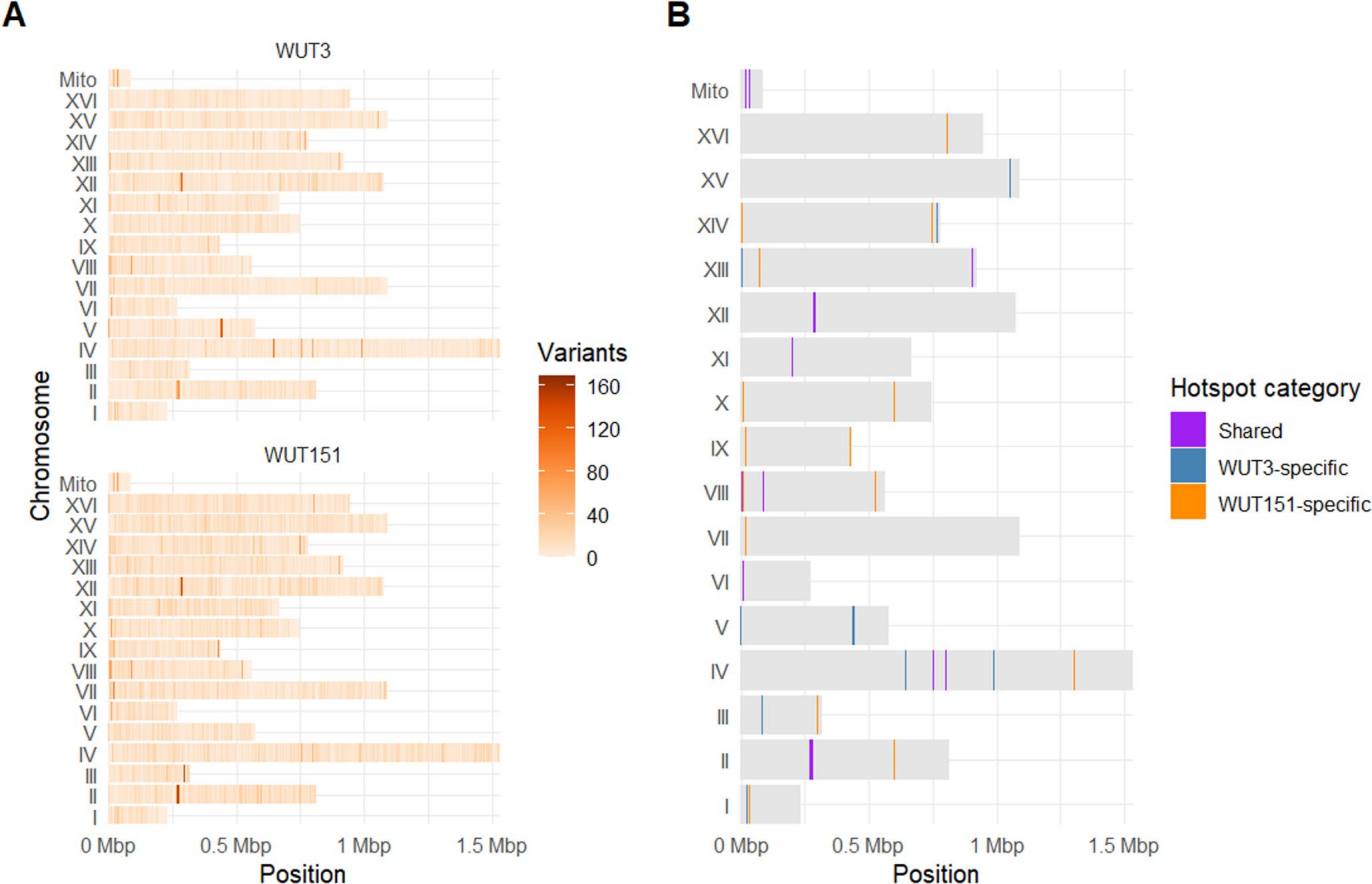
Variant distribution on WUT3 and WUT151 chromosomes. **A**: variant density calculated in 10 kb blocks. **B**: regions with high variant density (99th percentile). Shared hotspots indicate regions with high variant density in both genomes. Hotspot at chromosome II contains: *GAL1*, *GAL7*, *GAL10*; hotspot at chromosome XII contains: *EMP49*, *GAL2*, and *SRL2*; hotspot at Mitochondrial sequence (Mito) contains: *COX1* and *COB*

The distribution of high and moderate variants was uniform across the chromosomes, with higher variant density at the subtelomeric regions (Fig. 3 A). In both strains, there were two wide regions with higher variant density: the 270–280 kb region of chromosome II and the 290–295 region of chromosome XII (Fig. 3B). The first region contains the *GAL* gene cluster, with multiple homozygous variants in *GAL1*, *GAL7*, and *GAL10*, whereas the second region contains *EMP49*, *GAL2*, and *SRL2*, which were disrupted. Smaller regions with a high variant rate were also detected in *NUM1* (chr IV), *AAD6* (chr VI), *PUM1*, and *PGM1* (both at chr XI). WUT3 and WUT151 had the highest variant rate per million base pairs in their mitochondrial sequences, with the *COX1* and *COB* regions carrying the highest variant densities (Fig. 2B). Variants were found primarily in intron sequences, for instance, *AI2*, *AI4*, and *AI5* in *COX1*, and *BI2* in *COB*.

To assess the potential impact of the identified variants on the probiotic profile of the WUT3 and WUT151, genes previously reported to contribute to beneficial traits in *S. boulardii* were further investigated. These included genes associated with antibacterial activity (*SDH1*, *WHI2*), adhesion (*FLO1*, *FLO5*, *FLO10*, *FLO11*), heat stress response (*HSP26*, *HSP42*, *HSP60*, *HSP78*, *HSP82*, *HSP104*, *HSP150*, *SSA1*), and salt stress tolerance (*ENA1*, *ENA2*). These findings were then combined with the structural variant analysis and are summarized in Table 5.

### Structural comparison of *de novo* assembled genomes

While genome mapping provided high statistical power for SNP detection based on raw read depth, it is inherently limited by reference bias and struggles to resolve complex structural rearrangements. Therefore, the WUT3 and WUT151 genomes were reconstructed for the first time using *de novo* assembly, with completeness of 97.7% and 99.2%, respectively, as assessed by BUSCO against the *Saccharomycetes* database (Cherry 2015). Each genome contained 17 contigs (16 chromosomes and 1 mitochondrial sequence), totaling 11.75 Mbp and 11.64 Mbp, respectively. In the whole genome alignment, WUT3 shared 99.43% of bases with S288C, while WUT151 shared 99.53% (Table 4). The global GC content of WUT genomes reached 38%, while the GC content of mitochondrial sequences was over 2 times lower. As a reference, the S288C genome has a global GC content of 38.15% and mitochondrial GC content of 17.11%. Consistent with yeast mitochondria features, their genome was enriched in tRNA-coding regions. Pseudogenes and hypothetical proteins accounted for 7.9% of all protein-coding sequences, which is slightly higher than the ~ 5% previously reported for the S288C reference genome (Dietrich et al. 2023). They were evenly distributed across chromosomes, with an increased frequency on chromosomes II, VIII, and IX (Fig. S3).

**Table 4:** General features of WUT3 and WUT151 genomes, based on Mumer4 report

Feature	WUT3	WUT151
Aligned bases	99.43%	99.53%
Unique alignments identity	99.26%	99.15%
Global GC content	38.15%	38.19%
Mitochondrial GC content	15.77%	15.91%
Gene density (gene/Mb)	529	532

**Fig. 4:**
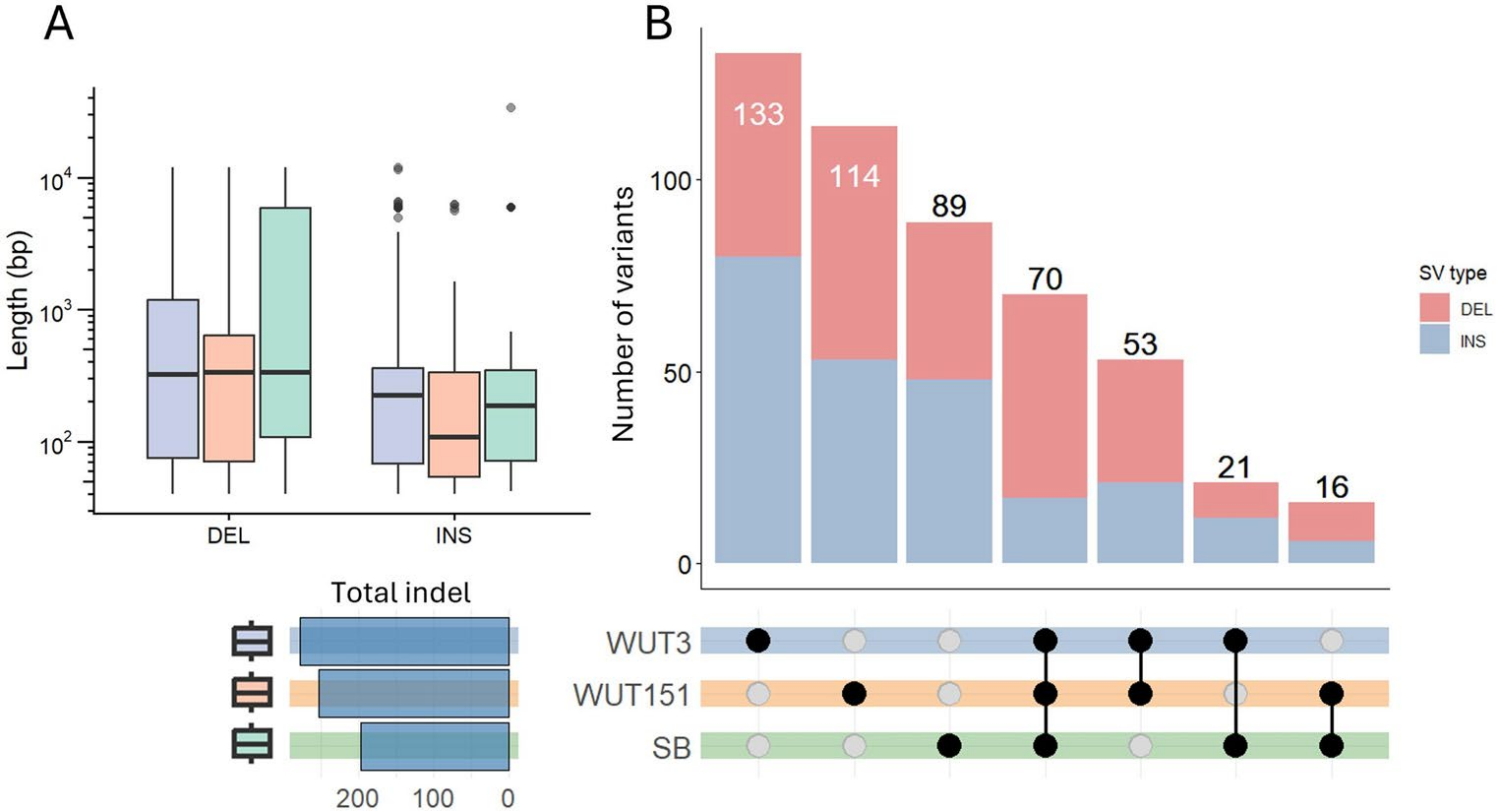
Characteristics of structural insertion (INS) and deletions (DEL) among WUT3, WUT151, and SB genomes. **A**: Length distribution of indels. The line represents the median, and the box represents the IQR. **B**: intersection of indes between WUT3, WUT151, and SB. SVs were considered common if they shared the same chromosome, start position, end position, and SV type

For the structural variant analysis, the *S. cerevisiae* var. *boulardii* (SB) CNCM I-754 genome was analyzed in parallel to identify potential variants that may differ from those of the probiotic strain. Due to insufficient data, the mitochondrial sequence was excluded from the *S. boulardii* dataset. WUT3 and WUT151 genomes were colinear with S288C, while SB had a rearranged terminal region of chromosome XVI, although this event was not explicitly classified as an inversion by the SV caller. Several contigs aligned in reverse orientation relative to the reference, which was interpreted as a consequence of contig orientation in the assembly rather than as evidence of independent large-scale inversion events (Fig. S4). The structural variant (SV) analysis revealed one large inversion (6 kbp) covering the entire *Tl1* element on WUT151 chromosome XIV and one small inversion (301 bp) covering the *Tl2* element on WUT3 chromosome II. Globally, WUT3 contained fewer deletions than WUT151 (150 and 159, respectively) and more insertions (132 and 98, respectively). SB contained fewer indels than WUT3 and WUT151 (Fig. 4). The median length of deletions was similar across the strains, whereas WUT151 showed a lower median length of insertions (108, IQR 282) compared to WUT3 (224, IQR 292) and SB (187, IQR 274) (Fig. 4 A).

The variant intersection analysis revealed that SB contains 89 unique indels absent in any of the WUT strains (Fig. 4B). Those SV may bring biological insights into their high probiotic potential. Moreover, 70 SVs were shared among all strains, which may contain modifications supporting the probiotic potential of WUT3 and WUT151. The importance of those SV and indels specific to SB and shared by all strains was examined using IGV.

**Fig. 5:**
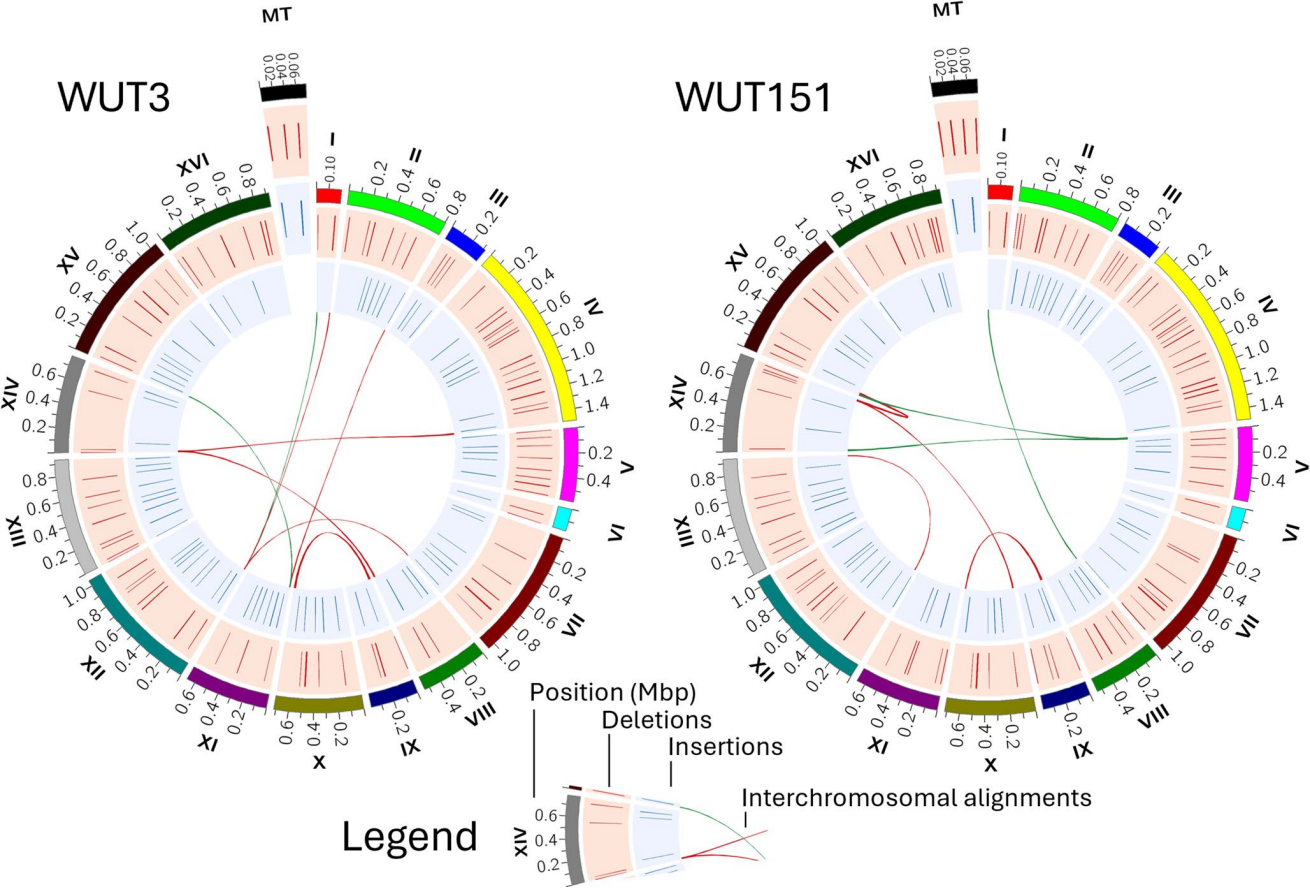
Structural variants of WUT3 and WUT151 genomes. Variants were determined using SVIM-asm. Red area – deletions, blue area – insertions. Links represent high-confidence interchromosomal alignments (quality > 20, length > 5000), colored by strand (green = forward, red = reverse). Inversions were not shown due to the low frequency and length

Most of the shared SVs were associated with retrotransposons (Ty1, Ty2), gag-polymerase pseudogenes (*YJR029W*, *YGR161C-D*), and long terminal repeats (Ty1LTR, Ty2LTR). These SV types comprised the longest observed deletions, exceeding 11 kbp (Fig. 5). Deletions involving transposable elements are common in yeast genomes and are generally not considered significant. Another large group of SVs was in subtelomeric or intragenic regions. In agreement with this distribution, the Circos plot based on minimap2 PAF alignments revealed several apparent interchromosomal matches, all confined to subtelomeric regions. Given the repetitive nature and high sequence similarity of these regions, such signals are unlikely to represent genuine translocations and are more reliably explained by homologous subtelomeric alignments.

After excluding SVs associated with retrotransposons, long terminal repeats, and ambiguous subtelomeric alignments, 45 genes remained as candidates for potentially relevant structural disruption. Further analysis focused on loci that may influence the probiotic potential, particularly genes involved in stress response (especially ion, osmotic, and thermal stress), adhesion, and cell wall organization (Pais et al. 2020). The *ENA* gene cluster is essential for yeast growth in salt stress conditions (Crespo et al. 2001). A wide deletion of *ENA1* and *ENA2* was identified in the WUT151 and SB genomes, whereas only *ENA1* was missing in the WUT3 genome. Analysis of the *FLO* gene family revealed poor mapping regions with low coverage. This led to an increase in variant calls that were not considered valid. Cell wall structure is one of the features that differentiate *S. boulardii* from *S. cerevisiae* (Ting et al. 2026). Indeed, the number of cell wall-related genes carried SV: *SED1*, *HSP150*, *PIR1*, *PIR3*, *TIR1*, *TIR3*, *DAN4*, *MNN4*, *WSC3*, and *CHS5*. Some of those genes have paralogues that remained unchanged and are therefore less likely to impact the phenotype: *SED1*–*SPI1*, *PIR1*–*PIR5*, *TIR3*–*TIR2*, and *WSC3*–*WSC2* (Table 5). *HSP150* and *PIR3* are also paralogues, but both contain structural indels in WUT3, WUT151, and SB genomes. There were two types of deletions in *HSP150* and two types of insertions in *PIR3*. Interestingly, WUT3 and WUT151 carried only one type of indel, whereas SB contained both. Products of those genes stabilize the cell wall structure under heat shock or oxidative stress, and null mutants show increased cell wall disruption (Yilmaz Kardas 2025). *MNN4* had significant deletions at the 3’ end of the CDS in all examined strains. Its paralogue, *MNN14*, did not have any structural variants, but WUT3 and WUT151 carried frame shift insertion of the TATA tandem sequence. This may reflect a real variant; however, the fact that both WUT strains contain the same repetitive insertion suggests it was due to a sequencing or base-calling artifact. Null mutants exhibit abnormal cell wall morphology, associated with reduced mannose content (Qin et al. 2025). Another mutation was detected in the *CHS5* gene, where WUT3, WUT151, and SB shared the same 105 bp deletion.

**Table 5:** Genes with variants potentially affecting probiotic-associated traits in WUT3, WUT151, and SB. Genes were classified as mutated when either a structural variant (SV) or a confident high-impact SNP was detected. “+” indicates a potentially disruptive mutation, “–” indicates no such mutation detected, “R” indicates that the detected mutation is likely functionally redundant due to the presence of a functional paralog, and “ND” indicates insufficient mapping quality for reliable assessment

Gene	Paralogue	Null phenotype impact	Variant status		
			WUT3	WUT151	SB
*ENA1*		Salt stress sensitivity	+	+	+
*ENA2*		Salt stress sensitivity	–	+	+
*SED1*	*SPI1*	Oxidative stress sensitivity	R	R	R
*FLO1*		Adhesion reduced	ND	ND	–
*FLO10*		Adhesion reduced	ND	ND	+
*FLO11*		Adhesion reduced	ND	ND	+
*HSP26*		Thermal sensitive	–	–	–
*HSP42*		Thermal sensitive	–	–	–
*HSP60*		Thermal sensitive	–	–	–
*HSP78*		Thermal sensitive	–	–	–
*HSP82*		Thermal sensitive	–	–	–
*HSP104*		Thermal sensitive	–	–	–
*SSA1*		Thermal sensitive	–	–	–
*HSP150*	*PIR3*	Cell wall stability reduction	+	+	+
*PIR3*	*HSP150*	Cell wall stability reduction	+	+	+
*PIR1*	*PIR5*	Cell wall stability reduction	R	R	R
*WSC3*	*WSC2*	Cell wall stability reduction	R	R	R
*MNN4*	*MNN14*	Cell wall morphology	R	R	R
*CHS5*		Cell wall morphology	+	+	+
*TIR3*	*TIR2*	Cell morphology	–	R	R

### Reference-based and *de novo* genome annotation

The assembled genomes provided a robust framework for analyzing structural variations. To further characterize the genetic composition of WUT strains, we performed genome annotation using a combination of reference-based transfer and *de novo* prediction tools.

First, genomic features from reference *S. cerevisiae* S288C were mapped onto both assemblies using the Liftoff tool. This approach enabled the identification of conserved genes and regions that exhibit substantial divergence from the reference. Gene transfer from the reference succeeded for over 96% of all annotations (Table 6). The features that failed to transfer from the reference were distributed equally between the strains, with approximately 32% unique to each strain and 38% shared. The WUT3`s and WUT151`s genomes lacked 230 and 226 features, respectively, that were present in the reference genome. At the protein-coding level, the WUT3 and WUT151 genomes contained 5,783 CDS and 5,790 CDS, respectively.

**Table 6:** Comparison of reference-based and de novo annotation outcomes

Strain	Liftoff CDS	Unmapped CDS	Transfer Success	AUGUSTUS CDS	Novel CDS	Prediction Success
WUT3	5783	230	96.2%	5349	13	92.4%
WUT151	5790	226	96.3%	5376	7	92.8%

To complement the reference-based annotation, *de novo* gene prediction directly from the assembled genomes was employed. This approach allowed for the identification of potentially novel coding sequences specific to these probiotic strains. Given their distinct geographical origins, those unique sequences may contribute to the genetic variability and specialized functional traits of WUT3 and WUT151. The AUGUSTUS tool predicted 5,349 CDS in WUT3 and 5,376 CDS in WUT151, with success rates over 92%, and revealed 13 and 7 CDS, respectively, that were not present in S288C (Table 6).

All novel CDS were annotated using NCBI BLAST with at least 90% identity. Analysis revealed that 4 of the WUT3 CDS corresponded to duplicated genes across different chromosomes. Out of 11 unique novel CDS sequences, 4 were identified only in the WUT genome, 3 were identified only in the WUT151 genome, and another 4 were shared by both strains (Table 7). Although we successfully annotated new genes, they were poorly described in the NCBI database, and none had an assigned function in either the NCBI or InterPro databases.

**Table 7:** Annotation of the novel CDS absent from the S288C reference, based on NCBI BLAST hits. E-values reported as 0.0 were below the numerical precision of BLAST. The “Shared” column indicates whether a given CDS was strain-specific or detected in both WUT strains

Strain	Shared	NCBI ID	Chromosome	E-value	Identity
WUT3	−	CAC9916144.1	XIII, XVI	10^− 68^	96.2%
	−	CAD6610728.1	IV	10^− 98^	99.3%
	−	EGA59958.1	XIII, XVI	10^− 66^	100%
	−	GMC39638.1	X, XV	10^− 118^	97.7%
	+	KAJ1055332.1	II	10^− 44^	98.7%
	+	CAI5281764.1	V	0.0	93.8%
	+	EDZ68592.1	V, XIII, XVI	< 10^− 104^	> 98.7%
	+	GAA5351211.1	MT	10^− 140^	98.1%
WUT151	−	EGA56435.1	XVI	0.0	93.8%
	−	KAJ1051632.1	VI	10^− 101^	100%
	−	GES72241.1	VI	10^− 65^	99.0%
	+	KAJ1055332.1	II	10^− 44^	97.3%
	+	CAI5281764.1	V	0.0	91.4%
	+	EDZ68592.1	V	10^− 150^	99.1%
	+	GAA5351211.1	MT	10^− 140^	98.1%

### Functional comparison of *de novo* assembled genomes

Since the *de novo* predicted genes lack functional descriptions in the databases, the subsequent functional analysis focused on the genes successfully transferred from the S288C reference. This approach allowed us to determine whether the missing features were randomly distributed or resulted from the targeted loss of specific function or gene families.

The ShinyGO functional annotation of the mismatched genes revealed high overrepresentation of viral genes, FE (fold enrichment) > 14.5, accounting for 19% of WUT3 and 30% of WUT151 missed genes. This likely explains their absence in the assemblies. The remaining non-viral genes were mostly uncharacterized or of unknown function. Still, significant overrepresentation of DUP/COS family genes was identified for both strains, reaching FDR (false discovery rate) < 10^− 12^ and FE > 19.0 (Table 8). Notably, the WUT3 genome lacked all genes associated with asparaginase activity and purine metabolism pathways (FE > 38), in contrast to WUT151, which contained those genes.

**Table 8:** Gene Ontology terms (ShinyGO) with the highest Fold Enrichment (FE) among non-viral genes missing in WUT3 and WUT151 strains

Strain	FDR	Genes	Pathway Genes	FE	GO terms
WUT3	2.2 × 10⁻⁶	5	5	38.7	Asparaginase, N-terminal
	2.2 × 10⁻⁶	5	5	38.7	Purine metabolism
	2.6 × 10⁻¹¹	12	23	20.2	Yeast membrane protein DUP/COS
	4.4 × 10⁻¹¹	12	24	19.3	DUP family
	6.0 × 10⁻¹³	18	56	12.4	Mixed 1
	6.5 × 10⁻¹⁷	24	79	11.7	Mixed 2
	2.7 × 10⁻¹⁸	27	94	11.1	Mostly uncharacterized 1
	1.4 × 10⁻¹⁶	34	196	6.7	Mixed 3
	1.5 × 10⁻⁶	15	89	6.5	MFS transporter superfamily
	4.3 × 10⁻⁷	19	136	5.4	Mixed 4
WUT151	1.9 × 10⁻⁹	7	8	41.5	Mostly uncharacterized 2
	2.3 × 10⁻¹¹	10	16	29.7	Mostly uncharacterized 3
	3.1 × 10⁻¹¹	11	22	23.7	Mostly uncharacterized 4
	9.2 × 10⁻¹¹	11	24	21.7	DUP family
	1.9 × 10⁻⁹	10	23	20.6	Yeast membrane protein DUP/COS
	1.1 × 10⁻²⁰	22	56	18.6	Mixed 1
	2.6 × 10⁻²⁶	29	79	17.4	Mixed 2
	4.1 × 10⁻¹²	19	89	10.1	MFS transporter superfamily
	4.4 × 10⁻²²	36	196	8.7	Mixed 3

Aberrations: Mixed 1 - Mixed, incl. maltose metabolic process, and asparaginase, n-terminal, Mixed 2 - Mixed, incl. maltose metabolic process, and mannose transmembrane transporter activity, Mixed 3 - Mixed, incl. active transmembrane transporter activity, and oxidoreductase activity, acting on the c, Mixed 4 - Mixed, incl. amino acid transport, and import into cell, Mostly uncharacterized 1 - Mostly uncharacterized, incl. maltose metabolic process, and mannose transmembrane transporter activity, Mostly uncharacterized 2 - Mostly uncharacterized, incl. anchored component of membrane, Mostly uncharacterized 3 - Mostly uncharacterized, incl. uncharacterized protein *YOR389W*-like, and *GFD2*/*YDR514C*-like, Mostly uncharacterized 4 - Mostly uncharacterized, incl. uncharacterized protein yor389w-like, and butanoate metabolism

Although several glucose transporter genes, specifically hexose symporters (HXT), were not mapped, both WUT strains can grow on glucose as a carbon source. This is likely due to the high functional redundancy among the HXT family members. *HXT6*, *HXT15*, and *HXT16* were absent in both WUT strains; *HXT8* and *HXT13* were not found in WUT3, while *HXT1*, *HXT4*, and *HXT7* were not present in the WUT151 genome.

**Fig. 6:**
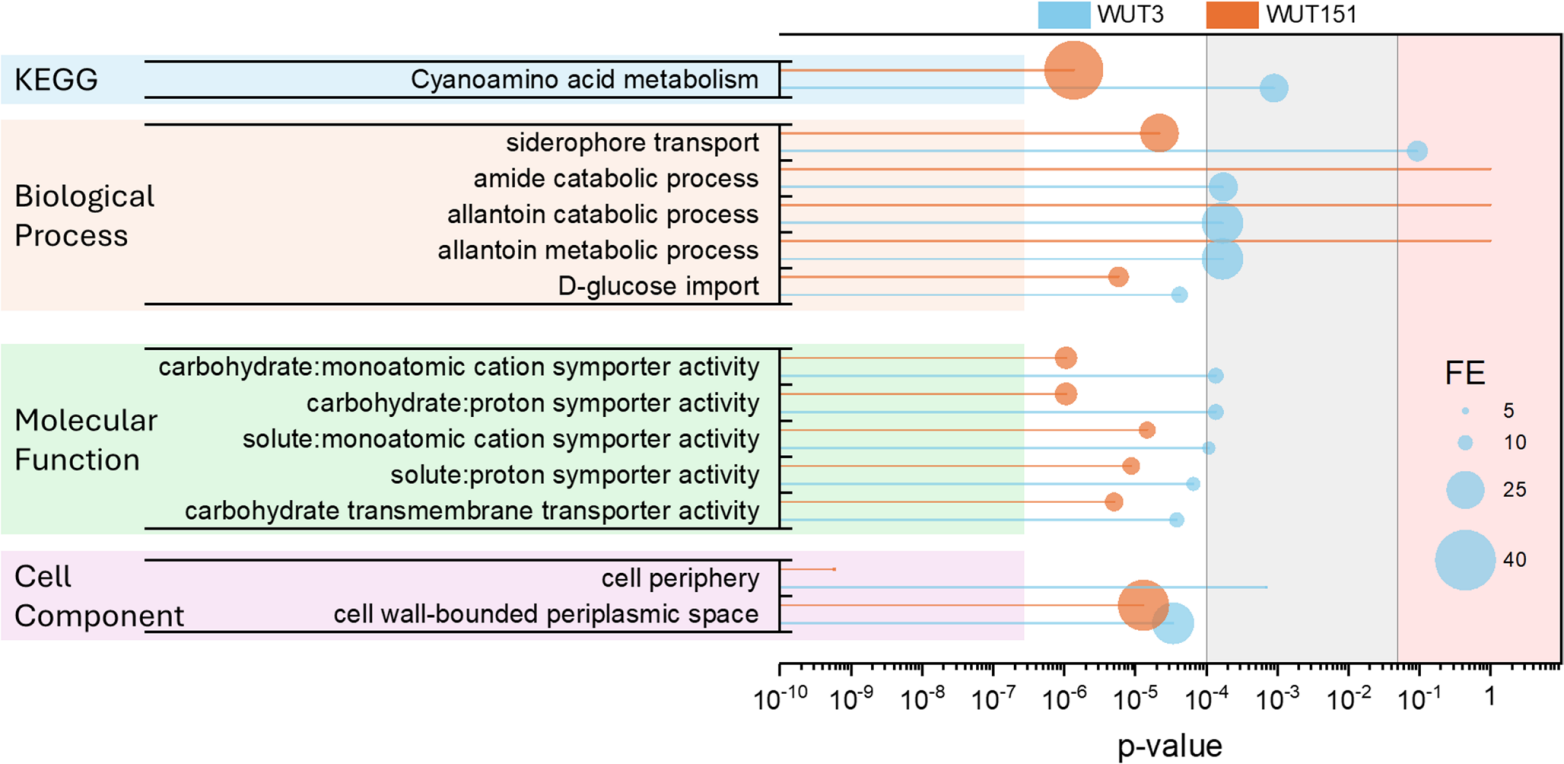
Gene Ontology enrichment analysis (g: Profiler) of the unmapped genes in WUT3 and WUT151 strains. The figure presents the top 10 functional terms for each strain, ranked by FDR. Unshaded area – high significance (FDR ≤ 10^− 4^). Gray area – moderate significance (10^− 4^ < FDR ≤ 0.05). Pink area – not significant (FDR > 0.05). FE – fold enrichment

In the Molecular Function category, both strains showed a deficiency in symporter-related genes. This was more significant for WUT151, which reached a lower FDR with greater FE (Fig. 6). Among the unmapped genes, we found a notable overrepresentation of those associated with the cell periphery, particularly the cell wall-bound periplasmic space (FDR < 10^− 4^, FE > 40).

To complement the initial findings, a second enrichment analysis was performed using g: Profiler. This approach focused specifically on yeast genes and completely disregarded the contribution from viral and transposable elements (Fig. 6). WUT151 showed a significant deficiency in genes involved in cyanoamino acid metabolism (FE > 25, FDR = 10^− 5^), as did WUT3, although with lower statistical significance (FDR = 10^− 3^). WUT3 genome lacked genes involved in amide and allantoin catabolism, while WUT151 was significantly deficient in genes related to siderophore transport. Notably, neither strain contained any of the four *ASP3* gene copies. As a result, the “asparaginase” GO term was significantly enriched (FE 21.6 for WUT3 and 26.9 for WUT151), which corresponds to the “cyanoamino acid metabolism” pathway in the KEGG database (Fig. 6).

In summary, the genes present in the reference genome but absent from the WUT genomes appear to be environmentally dependent.

### PCR verification of *HXT* and *ASP3* genes

In silico analysis revealed the absence of certain genes in the WUT genomes, which may significantly influence yeast metabolism. Most notably, WUT strains lacked the whole *ASP3* gene family and specific hexose transporters (*HXT*). To validate these computational findings, a polymerase chain reaction (PCR) was performed on genomic DNA extracted from WUT and reference strains. Representative genes from the key metabolic categories were selected for experimental verification.

**Fig. 7:**
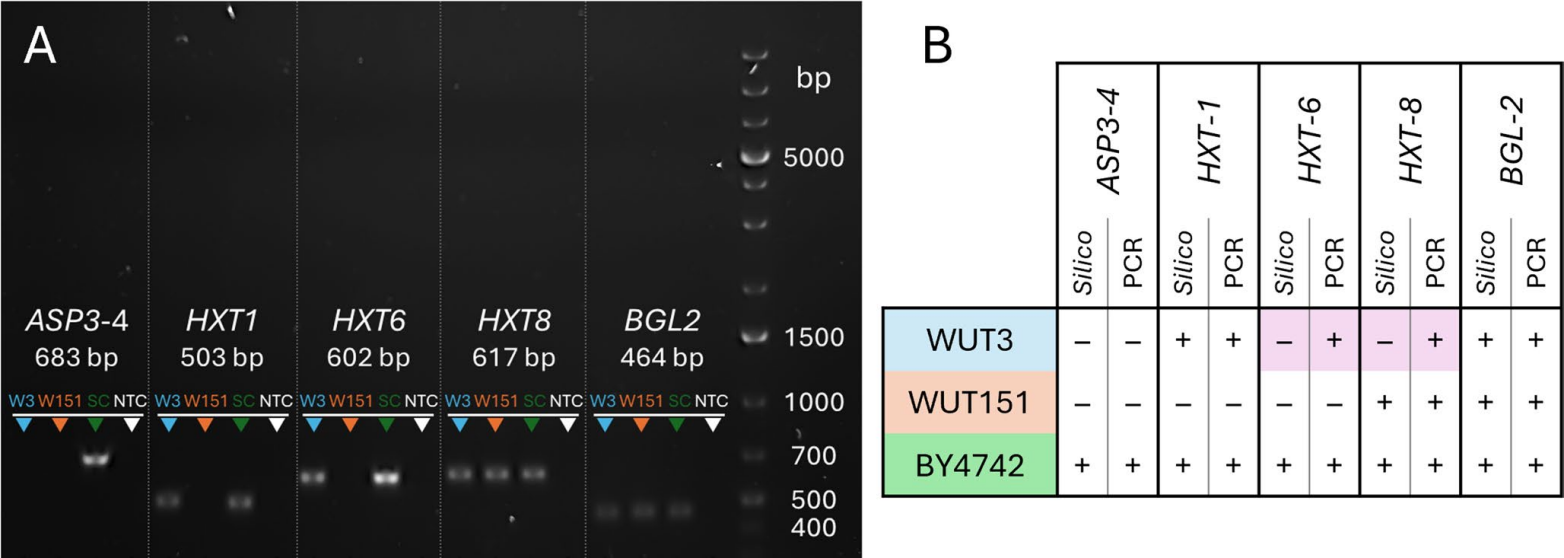
Validation of in silico gene presence predictions via PCR. **A**: PCR amplification of *ASP3* and *HXT* genes in WUT strains, with *BGL2* as a positive control. **B**: Comparison of in silico predictions and experimental PCR results (discrepancies between methods are highlighted). W3 – WUT3; W151 – WUT151; SC – *S. cerevisiae* BY4742 (derivative of S288C); NTC – no template control

The absence of the *ASP3-4* gene in WUT3 and WUT151 was confirmed experimentally by the PCR (Fig. 7). Similarly, the absence of the *HXT1* gene in the WUT151 genome was verified. However, the analysis of other hexose transporters revealed discrepancies between in silico and PCR results. *HXT6* was detected via PCR in WUT3 and SC, despite its absence in both WUT assemblies. This gene was in a poorly covered region of chromosome IV, which may explain why it was omitted by the assembler. Besides, *HXT7*, which differs by only 3 nucleotides from *HXT6*, was detected in the WUT3 genome. Furthermore, *HXT8* was identified in all strains via PCR, despite this gene being absent from the WUT3 assembly, even though the corresponding genome region exhibited high coverage (DP > 30) in the raw reads. In the reference strain, the chromosome X region contains a gene cluster: *HXT9*–*IMA5*–*HXT8*. It was retained in the WUT151 assembly, whereas in WUT3, *IMA5* was replaced with *IMA3* (from chromosome IX), and *HXT8* was replaced with *ENB1* (from chromosome XV). Progressive Mauve alignment further confirmed that those regions are highly differentiated, consistent with the dynamic nature of subtelomeric regions (Loegler et al. 2025). To further investigate the presence of *HXT8* in the WUT3 genome and resolve the discrepancies, an additional BLAST search was performed using the *HXT8* CDS sequence. The most significant hit was identified on chromosome IV (363,796–365,335), with 90% coverage and 74% identity. However, this region had previously been annotated by Liftoff as the *HXT7* gene, which showed superior alignment metrics (100% coverage and 97% identity). Due to the high sequence similarity between *HXT7* and *HXT8*, the PCR products had nearly identical size, making it challenging to distinguish between those two paralogues.

### Comparative CDS analysis across *S. cerevisiae* WUT3, WUT151, S288C (SC) strains, and *S. cerevisiae* var. *boulardii* (SB)

Our earlier studies demonstrated that WUT strains exhibit probiotic properties similar to those of the widely used yeast probiotic *S. cerevisiae* var. *boulardii* (SB)(Gryciuk et al. 2026). To investigate whether the probiotic nature is associated with specific genomic features that might be absent or modified in WUT strains, a comparative analysis of their coding sequences (CDS) was performed. This was achieved through three sets of pairwise comparisons: WUT3 versus WUT151, each WUT strain versus the reference S288C (SC), and each WUT strain versus SB. This allowed us to evaluate the presence of unique genes and the conservation of sequences within shared CDS.

**Fig. 8:**
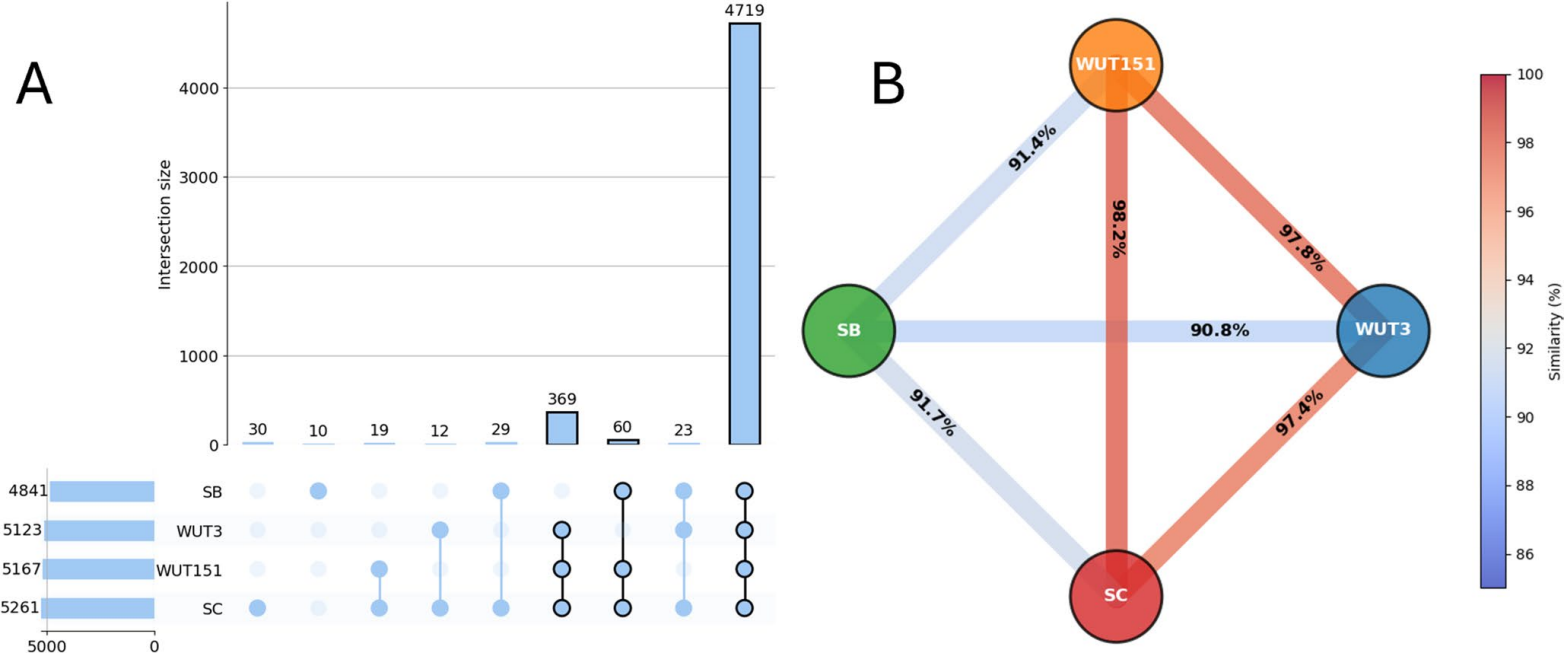
WUT3, WUT151, reference *S. cerevisiae* (SC), and *S. cerevisiae* var. *boulardii* (SB) genomes comparison based on the gene IDs. **A**: Upset plot, showing unique and shared gene counts across the groups. Groups with > 50 genes were bolded. **B**: Network graph, representing the Jaccard index between the strains (one-to-one comparison)

As many as 4719 genes (90%) were shared among all tested genomes (Fig. 8 A). WUT3, WUT151, and SC had 369 unique genes absent in SB, while SB had only 10 unique genes absent in any other genomes. The pairwise comparisons revealed that the SC and SB strains show a high, though not complete, similarity of 91.7% (Fig. 8B). Against this background, both WUT3 and WUT151 are more similar to the reference strain SC than to SB, with 97.4% and 98.2% vs. 90.8% and 91.4%, respectively. The overall Jaccard score for all 4 strains reached 89.53%.

Analysis confirmed that the tested yeasts have a similar gene profile. Therefore, we took a closer look at the differences in the protein or DNA sequences of shared genes. The translated CDS alignment (amino acid sequences) is called protein-level analysis, and the DNA alignment (nucleotide sequences) is called gene-level analysis.

At the protein level, we observed 5.6% ± 0.6% sequences with SIM (similarity) < 80% and 12.1% ± 1.6% sequences with ID (identity) < 80%, while in gene analysis, we identified 3.3% ± 1.0% and 11.6% ± 1.6% alignments, respectively (Fig. S5A, C). Interestingly, the number of perfect scores (SIM = 1.0) was almost 2 times higher at the protein level than at the gene level (29.6% ± 2.1%, 15.1% ±1.8%). The median SIM and median ID in each comparison were 0.99. Nevertheless, a population with lower scores was also observed, which appeared as a left-sided tail in the cumulative plots (Fig. S5B, D).

Overall, WUT3 and WUT151 displayed substantially fewer poorly aligned genes compared to SC (57 ± 4) than to SB (268 ± 2). This should be considered when interpreting both the absolute and relative number of genes assigned to a Gene Ontology (GO) term.

**Fig. 9:**
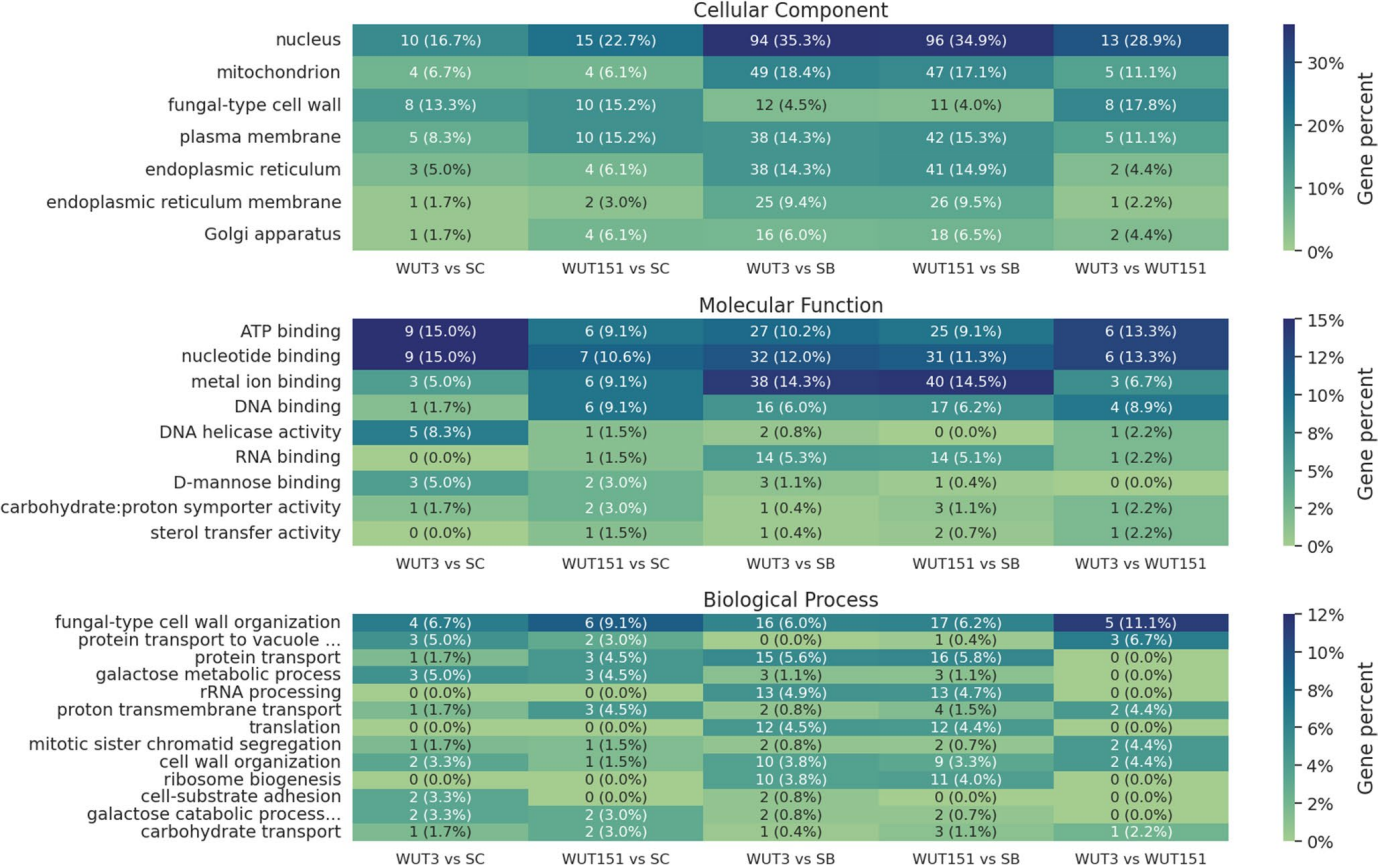
Heatmap shows the most frequently represented Gene Ontology (GO) terms in each comparison at the DNA level, separately for the three GO categories. Color intensity indicates the percentage of genes associated with each term, while numbers in the cells represent the absolute gene counts and their percentages

Within the cellular component category (Fig. 9), most low-aligned genes were associated with the nucleus (27.7% ± 3.6%). WUT3 and WUT151 showed fewer differences relative to the SC (10 and 15 genes, respectively) than to the SB (94 and 96 genes, respectively). A similar pattern was observed for the mitochondrion GO term.

Although WUT strains exhibited a higher percentage of low-scored genes associated with the fungal-type cell wall than SC strain, the absolute number of such genes was notably lower than in the SB (Fig. 9). *GAL1* (a nuclear gene) showed substantial divergence in both WUT vs. SB and WUT vs. SC comparisons, whereas SC and SB were highly similar. Likewise, only minor differences were observed between WUT3 and WUT151 (Table 9).

**Table 9:** Similarity score (SIM) of highly divergent genes in WUT strains compared to *S. cerevisiae* var. *boulardii* (SB). Genes with SIM below 0.5 in WUTs vs. SB comparisons were shown. Comparative data for the *S. cerevisiae* S288C (SC) reference are provided for context

GO term	Gene	SC SB	WUT151 SB	WUT151 SC	WUT3 SB	WUT3 SC	WUT3 WUT151
nucleus, nucleotide binding	*GAL1*	1.0	0.448	0.448	0.449	0.449	0.999
metal ion binding	*EMP46*	0.99	0.313	0.309	0.313	0.306	0.996
fungal-type cell wall	*DAN4*	0.955	0.0	0.0	0.0	0.0	1.0

Most differences in CDS nucleotide sequences were associated with the binding molecular functions (Fig. 9). WUT151 showed greater similarity to SC in ATP binding, nucleotide binding, and DNA helicase activity, whereas WUT3 was more similar to SC in metal ion binding and DNA binding. The most prominent differences observed between WUT strains and SB were in genes associated with metal ion binding, which accounted for more than 14% of all low-scoring sequences. Among ATP- and nucleotide-binding functions, *GAL1* was again the most disrupted gene. The greatest divergence among metal-ion-binding proteins was observed in *Emp46* (Table 9).

In the biological process category, the highest number of poorly aligned genes was associated with fungal-type cell wall organization (Fig. 9). Although WUT strains showed a higher relative proportion of these CDS when compared to SC, the absolute number of these genes was nearly half that identified in the SB. Additionally, SB differed more strongly from both WUT strains in genes associated with the GO term ‘cell wall organization’. Genes involved in transport also constituted a substantial proportion of low-scoring alignments. Among genes associated with fungal-type cell wall, *DAN4* showed the strongest sequence divergence (Table 9).

To determine whether nucleotide variations lead to altered protein products, a secondary analysis was conducted at the protein level (amino acids). This approach is biologically essential, as even a single nucleotide polymorphism can significantly impact protein structure and function. Furthermore, cross-validation of nucleotide and amino acid sequences enables more robust data interpretation, reducing the risk of sequencing artefacts in which a single base error can misrepresent the actual amino acid sequence.

**Fig. 10:**
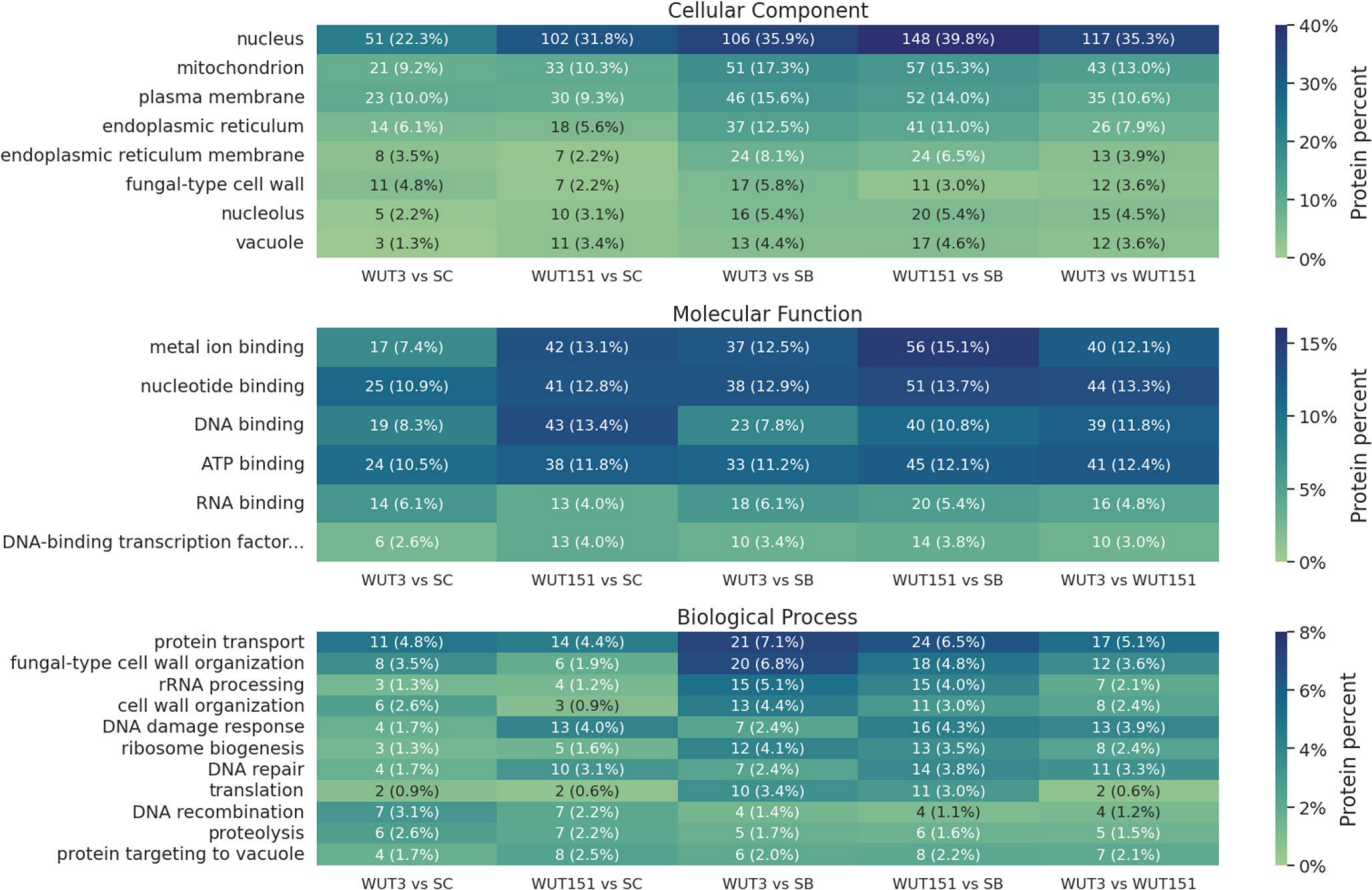
Heatmap shows the most frequently represented Gene Ontology (GO) terms in each comparison at the protein level, separately for the three GO categories. Color intensity indicates the percentage of genes associated with each term, while numbers in the cells represent the absolute gene counts and their percentages

WUT strains exhibited a comparable number of low-scoring proteins to SC and SB, averaging 294 ± 25 sequences. The largest number of weakly aligned proteins was associated with the nucleus GO term, representing 32.9% ± 5.9% of all low-scored proteins across comparisons (Fig. 10).

The in-depth exploration of nucleus-related proteins revealed that, among 203 unique proteins assigned to this category, 28 were involved in the regulation of DNA-templated transcription. WUT3 and WUT151 were more similar to SC than to SB in nuclear proteins, with 13.6% and 8.0% fewer low-alignment cases, respectively. The most disrupted proteins within the nuclear GO term were Gat1 and Tfc1, for which none of the WUT strains reached a SIM value above 0.5 (Table 10). Importantly, the gene sequences encoding those proteins did not differ substantially between SC and SB.

**Table 10:** Similarity score (SIM) of highly divergent proteins in WUT strains compared to *S. cerevisiae* var. *boulardii* (SB). Proteins with SIM below 0.5 in WUTs vs. SB comparisons were shown. Comparative data for the *S. cerevisiae* S288C (SC) reference are provided for context

GO term	Protein	SC SB	WUT151 SB	WUT151 SC	WUT3 SB	WUT3 SC	WUT3 WUT151
nucleus, binding proteins	Gat1	0.898	0.000	0.008	0.000	0.007	0.424
nucleus	Tfc1	0.743	0.267	0.357	0.256	0.344	0.358
plasma membrane, cell wall organization	Yps6	0.986	0.187	0.190	0.111	0.103	0.010
plasma membrane	Psr1	0.984	0.334	0.334	0.338	0.334	0.311
cell wall organization	Ecm12	0.000	0.000	0.126	0.000	0.057	0.066

Within the cellular component category, mitochondrial and plasma membrane proteins also constitute a substantial fraction of low-scored proteins (Fig. 10). Following a similar trend to the ‘nucleus’ category, those proteins were more prevalent in WUT vs. SB comparisons (approx. 15.6%) than in WUT vs. SC comparisons (approx. 9.7%). Differences were also observed between the two WUT strains. The most disrupted proteins in this category were Yps6 and Psr1, both of which retained high similarity between SC and SB (Table 10).

The molecular function (MF) category was dominated by binding proteins (Fig. 10). WUT151 showed the largest divergence among the metal ion-binding proteins, accounting for 15.1% of low-scored proteins relative to SB and 13.1% relative to SC. WUT3 exhibited a higher similarity to SC in ion-binding proteins (7.4% low-scored) but still differed from SB (12.5%). In contrast, WUT3 showed the lowest similarity to both SC and SB among nucleotide-binding proteins, accounting for 10.9% and 12.9% of weakly aligned proteins, respectively. WUT3 also differed from WUT151 in the nucleotide-binding proteins (13.3%). The most disrupted protein within the functional category was Gat1 (Table 10).

In the biological process category, the largest differences were associated with protein transport, cell wall organization, and rRNA processing (Fig. 10). Again, a higher number of low-scored proteins was observed in the WUT vs. SB comparison than in the WUT vs. SC. WUT151 contained more poorly aligned proteins than WUT3 in processes associated with DNA damage response and DNA repair. Interestingly, none of the *S. cerevisiae* strains (WUT and SC) showed changes in proteins related to translation, whereas over 3% of the SB low-scored proteins were assigned to this process. Among the lowest-scoring proteins in the cell wall organization category were Ecm12 and Yps6 (Table 10). Ecm12 is particularly noteworthy, as its sequence differed across all strains examined. We also identified several proteins that were absent in SB but retained in both WUT and SC genomes, including Nag1, Ack1, Mkz7, and Mtl1.

## Discussion

This study provides the first genomic characterization of two *S. cerevisiae* strains, WUT3 and WUT151, which show distinct probiotic potential (Gryciuk et al. 2026). Their origin from fermented dairy-associated niches further supports the view that milk products may serve as a reservoir of yeast strains with potential probiotic relevance (Lama et al. 2026). The research focuses on resolving their genomic architecture, investigating structural variants, and evaluating their genetic relationships with the laboratory reference strain *S. cerevisiae* S288C (SC) and the probiotic *S. cerevisiae* var. *boulardii* CNCM I-745 (SB).

Assembly completeness was consistent with quality thresholds from recent yeast genomic studies (Higgins et al. 2021; Marr et al. 2023). The WUT3 and WUT151 genomes reached the expected S288C genome size, with deviations of ± 3.5% (Nikodinoska and Moran 2024). Furthermore, the alignment of raw reads and *de novo* assembly confirmed a close relationship between WUT and SC (Jacobus et al. 2021).

The overall SNP profiles of WUT3 and WUT151 showed genome-wide variation, with only a small number of mutations predicted to have high-impact effects (Marr et al. 2023). The variant density was consistent across chromosomes, with a typical increase in the subtelomeric region. The global SNP per Mbp was moderate, yet similar to other reports on environmental *S. cerevisiae* isolates (Wei et al. 2007). Structural analysis revealed that the WUT3 and WUT151 genomes are colinear with the S288C. Long deletions and insertions dominated the structural variant (SV) calling. Most deletions were identified in the *Tyl1* and *Tyl2* regions. This profile is similar to that of the *S. boulardii* genome, which lacks transposable elements, compared to S288C (Pais et al. 2020). Prior to the main variant screening, aiming to identify probiotic-relevant changes, regions with an extreme number of high-impact variants were closely inspected. In particular, the *GAL* cluster was located at the edge of the ONT reads. While *GAL10*, *GAL1*, and *GAL7* retained high mapping quality and coverage, they are flanked by regions of poor coverage, suggesting that frame variants identified in those genes are sequencing artifacts. Similar issues were spotted in chromosome XII, where *EMP46*, *GAL2*, and *SRL2* were located at the read transition region. Therefore, those variants were not further considered significant.

Probiotic potential is a multidimensional subject in which variations in numerous genes can positively affect the yeast phenotype. While there is no direct evidence on which *S. boulardii* genes make it a sufficient probiotic, we focused on the most documented candidates (Pais et al. 2020). One of the main probiotic features is resistance to low pH, which allows yeasts to survive gastric acid. Most *S. boulardii* strains harbor *SDH1* F317Y and *WHI2* nonsense Ser287* variants, which were reported to reduce acid tolerance but, at the same time, to increase acetic acid production, which correlated with antimicrobial activity (De Carvalho et al. 2024). Neither strain contained variants in *SDH1*, but both harbored distinct missense SNPs in *WHI2* that were predicted to be potentially deleterious. Because these substitutions differ from the previously described nonsense variant, their functional consequences remain uncertain. Importantly, in our previous study, WUT3 and WUT151 displayed slightly increased resistance to gastrointestinal tract-like conditions compared to the *S. boulardii*, including low pH and bile salts, indicating that these variants are unlikely to compromise overall tolerance to such stress (Gryciuk et al. 2026).

In addition to low pH tolerance, probiotic strains should be resistant to osmotic stress. A recent study demonstrated that *ENA1* is an essential gene that confers resistance to high salt concentrations. Nevertheless, *S. boulardii ENA1-Δ0* strains survive in the gastrointestinal tract and retain their probiotic properties in the mouse model whilst exhibiting reduced virulence (Imre et al. 2026). Interestingly, a structural deletion of the entire *ENA1* locus was also detected in WUT3 and WUT151 strains. Furthermore, we found that the genomes of WUT151 and SB are disrupted in *ENA2*. While highly similar to *ENA1*, *ENA2* encodes a protein that is less effective at Na^+^ detoxification and is primarily associated with Li^+^ efflux (Ruiz and Ariño 2007). It can therefore be assumed that additional deletion of *ENA2* makes WUT151 and SB less resistant to high salt concentrations than WUT3. While this effect may not significantly reduce overall survival in the gastrointestinal tract, as with the *ENA1* deletion, it establishes WUT3 as a distinctive model among environmental isolates. The retention of intact *ENA2* is notable, as previous comparative analyses reported *ENA1*/*ENA2* depletion in most wild-type strains relative to S288C (Carreto et al. 2008). This gives WUT3 the potential to have superior adaptive advantages under specific environmental pressures.

Heat is another stress factor to which probiotics are exposed. Core heat shock proteins associated with heat stress (*HSP26*, *HSP42*, *HSP60*, *HSP78*, *HSP82*, *HSP104*, *SSA1*) did not contain significant variants, suggesting that the principal molecular machinery involved in thermal stress response is conserved in WUT3, WUT151, and SB (Yost and Lindquist 1991; Richter et al. 2010; Tiwari et al. 2015). Interestingly, all strains shared an identical structural deletion pattern in *HSP150* and *PIR3*. A similar observation has previously been reported for the film-forming *S. cerevisiae* strains, while the impact of these mutations on cell wall stability remains unclear (Kovács et al. 2008). Another structural deletion, conserved across all strains, was identified in the *CHS5* gene. Mutations in this gene are associated with reduced cell wall chitin content and increased thermal sensitivity when *GAS1* is also nonfunctional (Carotti et al. 2002). *GAS1* did not contain significant variants in WUT3, WUT151, or SB, indicating that the *CHS5* deletion does not affect their high-temperature tolerance, consistent with their phenotype.

Adhesion is another important aspect of probiotic yeast. The flocculation gene family has the greatest impact on adhesion. In particular, *FLO1*, *FLO5*, and *FLO10* were reported to increase cell-to-cell adhesion, while *FLO11* is responsible for cell-to-surface adhesion (Mohammadi and Saris 2023). Interpretation of *FLO* variants is challenging due to their subtelomeric location, which can affect sequencing reliability. While core Flo proteins were identified in the *S. boulardii* proteome, their sequences were disrupted in the genomic analysis (Khatri et al. 2017). Due to the same issues, we could not confirm the presence of *FLO1* and *FLO10* in WUT strains, whereas *FLO5* and *FLO11* contained multiple poor-quality variants. This suggests that variant analysis of *FLO* regions does not yield sufficient insights into adhesion features and that an experimental approach should be considered, as described in our previous study (Gryciuk et al. 2026).

According to the SGD, mutations in *COX1* and *COB* may reduce the colony size and impair growth on non-fermentable carbon sources (Cherry 2015; Gilea et al. 2021). Metagenomic analysis of *S. cerevisiae* mitochondrial sequences showed that mutations in the introns of *COX* and *COB* are highly strain-dependent and present in a mosaic pattern. Moreover, the presence of introns may be related to the origin of the particular isolate (Wolters et al. 2015). In our study, WUT3 and WUT151 were isolated from Turkey and Kyrgyzstan, which are relatively distinct locations. Nevertheless, both strains showed the same mutation pattern, with variants in *AI4* and *BI2*. This suggests that mitochondrial intron variation in WUT strains is strain-specific but not necessarily linked to their geographic origin.

The variant-based approach provided information on polymorphisms within conserved loci, but it was inherently limited to genes that could be aligned to the reference genome. Because phenotypic differences may also arise from variation in gene repertoire rather than from sequence polymorphism alone, we next assessed gene content across the analyzed genomes. This included comparing transferred annotations and *de novo* gene predictions to identify genes absent from the reference-based set and potentially novel strain-specific features.

*De novo* gene prediction revealed only a negligible number of additional candidate genes in the WUT genomes that were not represented in the *S. cerevisiae* S288C reference annotation. Among those, three showed strong similarity to sequences in the *S. boulardii* CNCM I-745 genome, with > 96% coverage and > 77% identity: GMC39638.1, KAJ1055332.1, and KAJ1051632.1. In the opposite comparison, fewer than 4% of S288C genes were absent from the WUT genomes. A substantial proportion of these missing genes corresponded to viral contaminants. Their absence is therefore unsurprising, as virus-related sequences are known to vary among strains of the same species (Nenciarini et al. 2024).

While most of the missing genes were poorly described in databases and were classified as “mixed-function” GO categories, several distinct trends emerged. The absence of certain DUP/COS family members was expected, as this is one of the largest and most variable multigene families in the *S. cerevisiae* genome (Despons et al. 2006). The COS subfamily (often referred to as *DUP380*), mostly localized in subtelomeric regions, is implicated in protein sorting and export. Functional studies have shown that loss of individual or multiple *COS* genes results in minor or no phenotypic effects, consistent with substantial functional redundancy within this family (MacDonald et al. 2015).

From a metabolic perspective, deficiencies in genes encoding enzymes of the cyanoamino acid pathway can slightly limit WUT strains’ ability to adapt to nitrogen-limited environments (Zhang et al. 2025). Another finding was the absence of the *ASP3* gene encoding a cell wall-bound asparaginase involved in the cellular response to nitrogen starvation and in adaptation to an environment rich in asparagine, such as fermented apple juice or beer. However, a recent in silico study revealed that over 93% (1563 of 1680) *S. cerevisiae* genomes do not contain even a single copy of *ASP3* (Coral-Medina et al. 2022). The probiotic *S. boulardii* CNCM I-745 also lacks the entire *ASP* gene family (Kaźmierczak-Siedlecka et al. 2020). Therefore, its absence in WUT strains is not unique, especially given their isolation from the dairy environment, where asparagine availability may not be a primary selective pressure.

The genomic analysis revealed differences in the hexose transporter (*HXT*) repertoire and in other genes involved in glucose import. Sugar transport is a key factor influencing yeast growth and fermentation, and *S. cerevisiae* has developed multiple, partially redundant mechanisms for sugar uptake (Donzella et al. 2023). Most observed *HXT* deficiencies concerned high-affinity or minor transporters (*HXT4*, *HXT6*, *HXT7*, *HXT8*, *HXT13*, *HXT15*, and *HXT16*), advantageous under glucose-limited conditions (Cherry 2015; Rizzo et al. 2017). Furthermore, a significant finding was the absence of selected low-, medium-, and high-affinity transporters in WUT151, including *HXT1*, *HXT4*, *HXT6*, and *HXT7* (Donzella et al. 2023). Given glucose-dependent *HXT* regulation and the predominant role of *HXT1* under high-glucose conditions, this transporter-loss pattern suggests reduced metabolic flexibility of WUT151 compared with WUT3 (Cherry 2015; Rizzo et al. 2017; Higgins et al. 2021). Nevertheless, a complete loss of glucose uptake requires simultaneous deletion of the major *HXT1*-*HXT7* transporter set (Özcan and Johnston 1999). Thus, the observed pattern is more likely to alter transport efficiency and flexibility. This suggests compensation by the remaining paralogues, consistent with *HXT* family redundancy (Özcan and Johnston 1999).

The discrepancies between in silico and PCR results in the *HXT* family highlight the superior power of genome sequence analysis when distinguishing highly similar paralogues. Due to the strong sequence homology within the *HXT* family, electrophoresis is insufficient to distinguish between loci that differ by only a few nucleotides, such as *HXT6* and *HXT7*. Accordingly, PCR initially suggested the presence of *HXT6* and *HXT8* in the WUT3, whereas detailed assembly analysis indicated that the amplified products most likely originated from a similar *HXT7* locus. This highlights that routine PCR-based screening can lead to erroneous conclusions about the metabolic potential of yeast strains. Using long-read sequencing and high-quality assembly, we successfully resolved these paralogous loci and corrected the misidentifications inherent in traditional methods.

The comparative analysis of CDS revealed subtle differences in the gene content and sequence similarity, indicating that the WUT strains are more closely related to the S288C reference strain than to the probiotic *S. cerevisiae* var. *boulardii*. Although SB and SC share 99% average nucleotide identity, our comparison showed that SB lacks approximately 10% of the genes annotated in SC (Pais et al. 2021). This discrepancy is larger than expected and may result from incomplete or inconsistent annotation of the SB genome, highlighting the need for further high-resolution NGS research to fill this gap.

From a biological perspective, protein alignment offers more meaningful insights into functional divergence, as protein activity depends directly on the amino acid sequence (Morrison 2006). However, next-generation sequencing occasionally introduces false nonsense mutations, which may artificially reduce protein similarity while DNA similarity remains high. Therefore, analyzing both protein and DNA levels provides a more reliable overview of strain differences.

Our sequence alignment analysis of shared CDS regions showed that only a minor proportion of proteins and genes differ across the genomes. At the DNA level, WUT strains were more similar to SC than to SB. This finding is consistent with previous comparative genomics studies, which show that, despite the overall high DNA sequence identity between *S. cerevisiae* and *S. cerevisiae* var. *boulardii*, yeasts differ in specific gene clusters (Duffey et al. 2025). Interestingly, this pattern was not reflected at the protein level, where WUT strains exhibited a similar number of low-scoring proteins in comparison with both SC and SB.

The largest divergence was observed in nuclear proteins and genes, especially those involved in transcriptional regulation, suggesting possible differences in regulatory capacity or gene expression control. However, several low-scoring loci appear to reflect technical rather than biological variation. *TFC1*, encoding an essential TFIIIC subunit, showed poor protein alignment despite > 99% DNA sequence identity and no high-impact read-supported variants, indicating a likely assembly or annotation artifact (Cherry 2015). Similarly, *GAL1* appeared divergent due to Liftoff-related CDS truncation, whereas full-length sequence inspection and InterProScan confirmed an intact coding region, consistent with galactose utilization by both WUT strains. Other low-scoring regulatory or DNA-binding genes, including *GAT1* and *EMP46*, should also be interpreted cautiously. *EMP46* lies in a poorly mapped region, making it difficult to exclude technical bias. In addition, its homolog *EMP47* showed no corresponding divergence. According to the SGD database, a single *EMP46* disruption is viable, whereas growth defects are observed only when both *EMP46* and *EMP47* are affected. While the enrichment of differences in nuclear and DNA-binding categories remains consistent with possible regulatory divergence, individual low-scoring loci require cautious interpretation due to potential assembly or annotation artifacts.

The protein-level comparison revealed low similarity of transport proteins. However, we did not observe any gene consistently disrupted across all WUT comparisons. The protein transport GO category covers over 390 proteins (Cherry 2015). Therefore, even if some differences were identified between strains, it is highly unlikely that they would impact any important features.

According to the biological process category, WUT strains were more similar to SC in terms of the cell-wall-related proteins. We have identified several SC genes that were absent in the SB genome. This is consistent with previous reports on differences in SC and SB cell wall structure (Ting et al. 2026).

## Conclusions

This study provides the first genomic characterization of the dairy-associated *S. cerevisiae* strains WUT3 and WUT151, both of which have been identified as having strong probiotic potential (Gryciuk et al. 2026). High-quality complete genomes were generated via *de novo* assembly and identified as closely related to the *S. cerevisiae* S288C reference. Overall sequence divergence was low, and the majority of detected SNPs and SVs were located in subtelomeric, repetitive, or other sequencing-challenging regions.

From the functional perspective, the core genomic background appears largely preserved in both WUT strains. In particular, the major heat shock protein genes were conserved, suggesting that the principal mechanism of high-temperature stress response remains intact. Interestingly, both WUT and probiotic reference strains shared a similar *HSP150* and *PIR3* deletion. The observed differences were limited to a small number of loci with uncertain functional significance or likely redundancy. Among the most notable findings, WUT151 lacked several hexose transporters, including *HXT1*, potentially limiting its ability to adapt to changing glucose concentrations.

Importantly, several of the observed genomic features were consistent with patterns previously reported in *S. cerevisiae* var. *boulardii*, suggesting that WUT3 and WUT151 may share selected genomic traits associated with probiotic potential. However, despite those similarities, both WUT strains remained more closely related to S288C at the CDS level. This indicates that probiotic potential in WUT3 and WUT151 is not reflected by a single, distinct probiotic genomic background, but rather by a limited set of dispersed features within an otherwise typical *S. cerevisiae* genome. Interestingly, the WUT3 strain has a unique profile that deviates from this pattern. By preserving an intact *ENA2* locus alongside probiotic-associated deletions, it represents a rare genomic compromise between environmental resilience and clinical safety. These findings also indicate that probiotic relevance cannot be inferred from genomic similarity alone but should be interpreted together with previously obtained functional data and, ultimately, confirmed by future clinical evaluation.

In summary, the analyzed genomes showed no major abnormalities and remained largely consistent with the typical *S. cerevisiae* genomic background. The identified differences were limited and may be linked to the probiotic potential of WUT3 and WUT151, supporting their further evaluation as candidate probiotic strains.

## Supplementary information

Below is the link to the electronic supplementary material.

Supplementary Material 1

Supplementary Material 2

## Data Availability

The raw reads from genome sequencing and assembled genomes are available in the European Nucleotide Archive (ENA) under accession no. PRJEB102465, https://www.ebi.ac.uk/ena/browser/view/PRJEB102465.Additional data regarding WUT3 and WUT151 strains can be found in the project repository, DOI: http://dx.doi.org/10.71724/9761-qn51.The codes are available online in the Zenodo repository. DOI: 10.5281/zenodo.19567034.
