# Supplementary material for: Genomic insights into the probiotic potential of dairy-associated Saccharomyces cerevisiae WUT3 and WUT151 strains
Source: Funct Integr Genomics. 2026 Jul 6;26(1):177. doi: 10.1007/s10142-026-01963-4 (PMC13333559; doi:10.1007/s10142-026-01963-4)
Supplement: Supplementary file 1 — Supplementary Material 1 [file 10142_2026_1963_MOESM1_ESM.docx]

^Functional & Integrative Genomics^

**Additional file 1: Supplementary figures**

Genomic Insights into the Probiotic Potential of Dairy-Associated *Saccharomyces cerevisiae* WUT3 and WUT151 Strains

Aleksander Gryciuk^1^*, Małgorzata Milner-Krawczyk^1^, Adrianna Skoneczna^2^, and Jolanta Mierzejewska^1^**

^1^ Laboratory of Microbiology and Bioengineering, Faculty of Chemistry, Warsaw University of Technology, Warsaw, Poland

^2^ Institute of Biochemistry and Biophysics, Polish Academy of Sciences, Warsaw, Poland

* Correspondence: aleksander.gryciuk.dokt@pw.edu.pl

** Correspondence: jolanta.mierzejewska@pw.edu.pl

| **A** |
| --- |
| 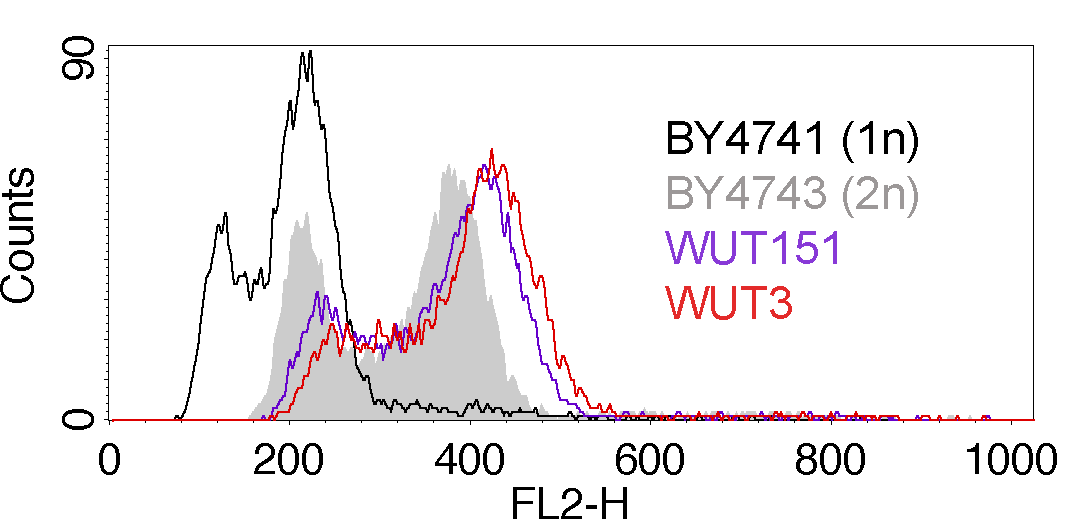 |
| **B** |
| 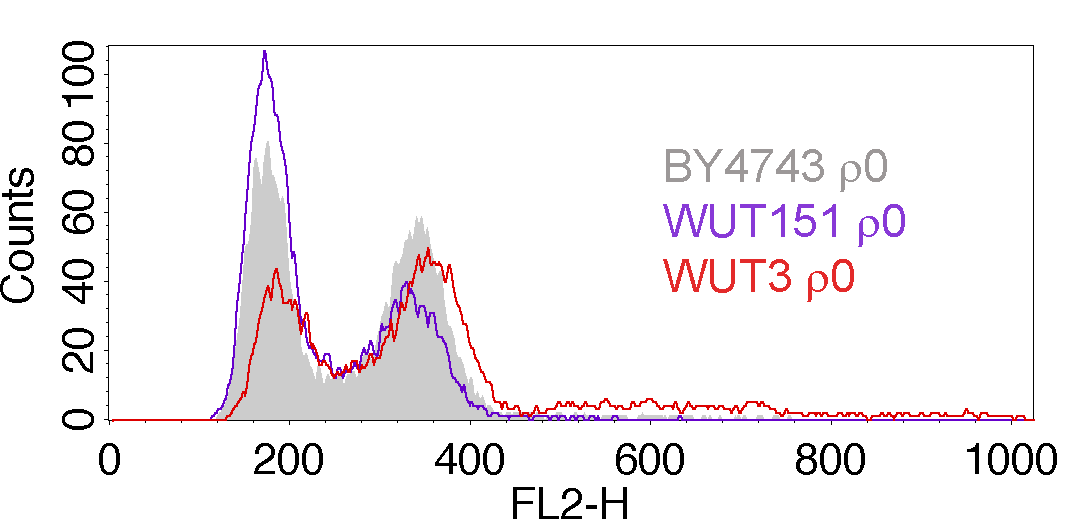 |

**Fig. S1** Flow cytometry analysis shows DNA content of WUT3 and WUT151, slightly increased compared to the *S. cerevisiae* reference, which likely relies on their higher mtDNA content.
**A -** Flow cytometric DNA content profiles of WUT3 and WUT151, with BY4741 as a 1n reference and BY4743 as a 2n reference. **B -** DNA content of WUT3 and WUT151 derivatives lacking mtDNA is shown relative to BY4743 ρ0 control.


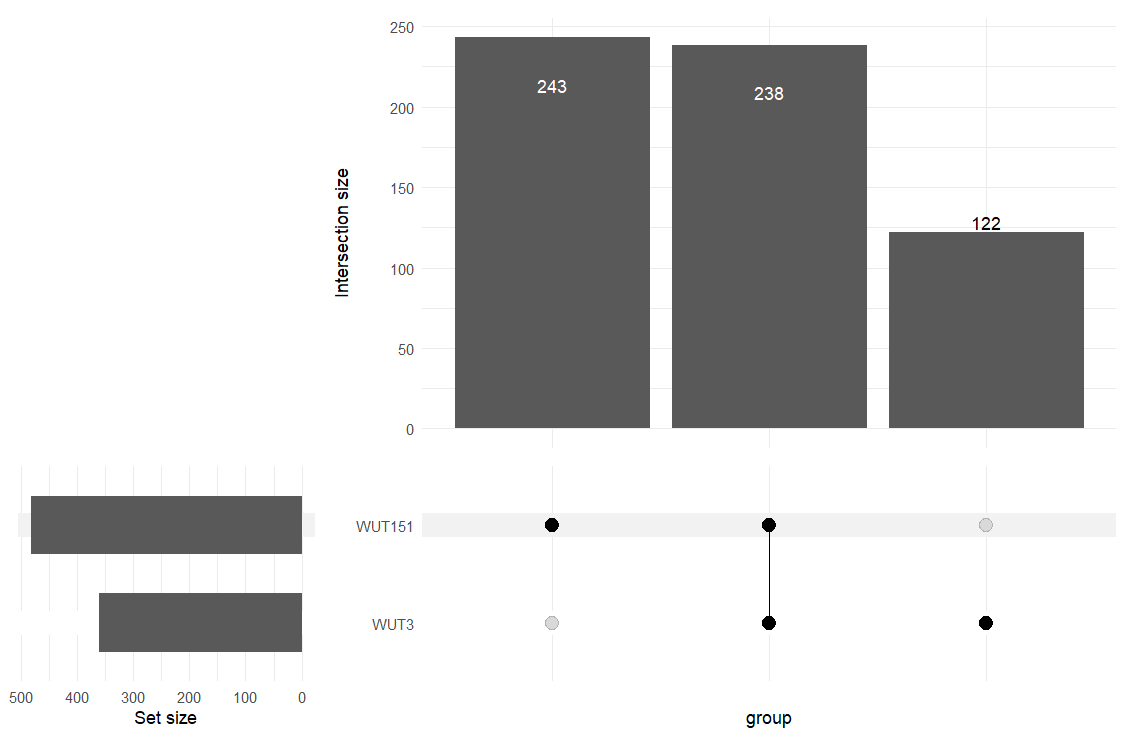


**Fig. S2** Upset plot showing the intersection of genes containing high-impact variants among the WUT3 and WUT151 strains.


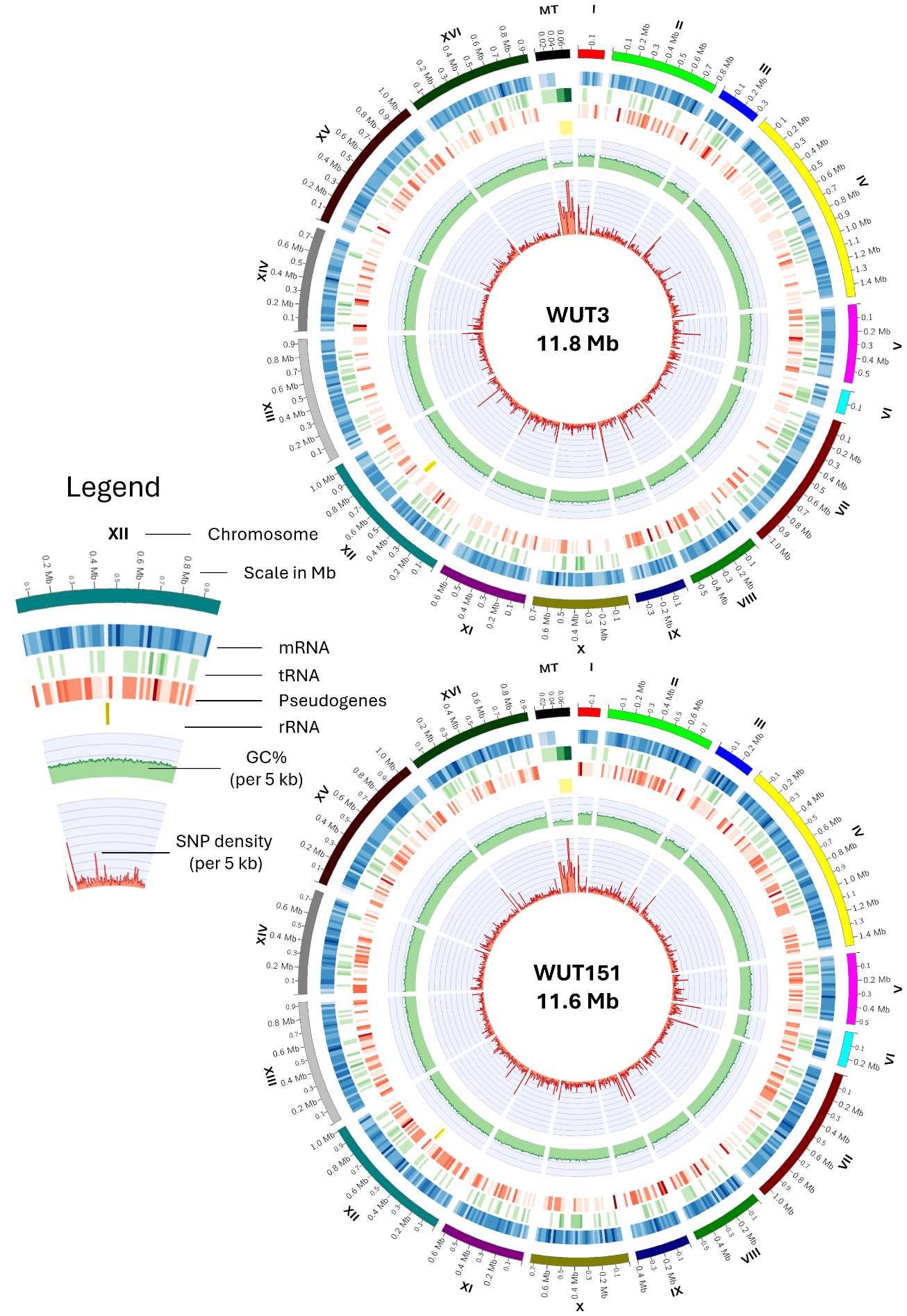


**Fig. S3** Map of the WUT3 and WUT151 genomes. From the outer circle: Position in Mb, protein coding DNA per 20 kb (blue), tDNA per 20 kb (green), DNA marked as pseudogene or hypothetical protein per 20 kb (orange), rDNA per 20 kb (yellow), GC content per 5 kb (green), SNP distribution per 5 kb (red).


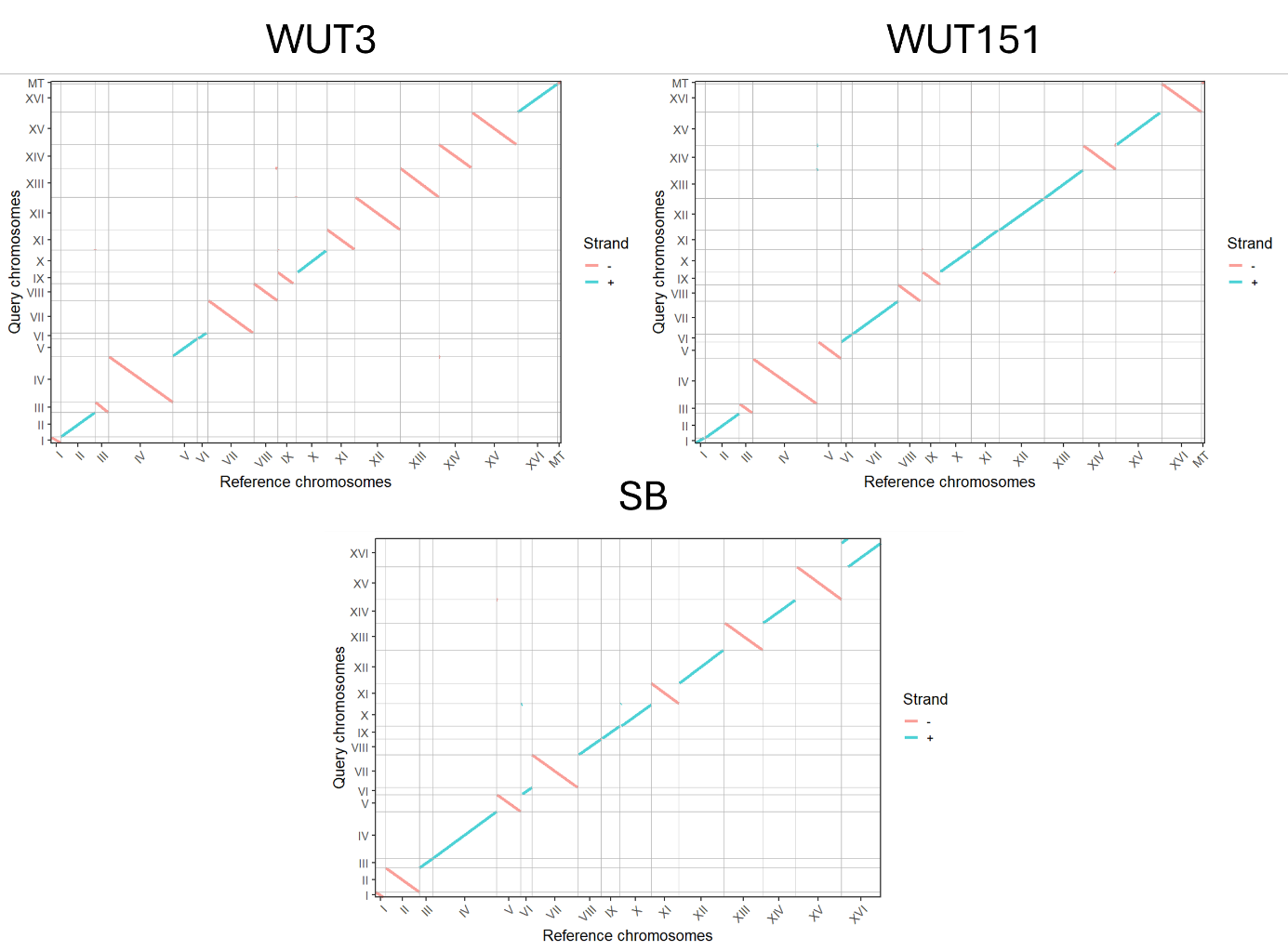


**Fig. S4** Dotplot of WUT3, WUT151, and SB genomes against the S288C genome, based on the minimap2 mapping. Blue lines represent colinear fragments, red lines represent reversed fragments. The mitochondrial sequence of SB was not included in the analysis.


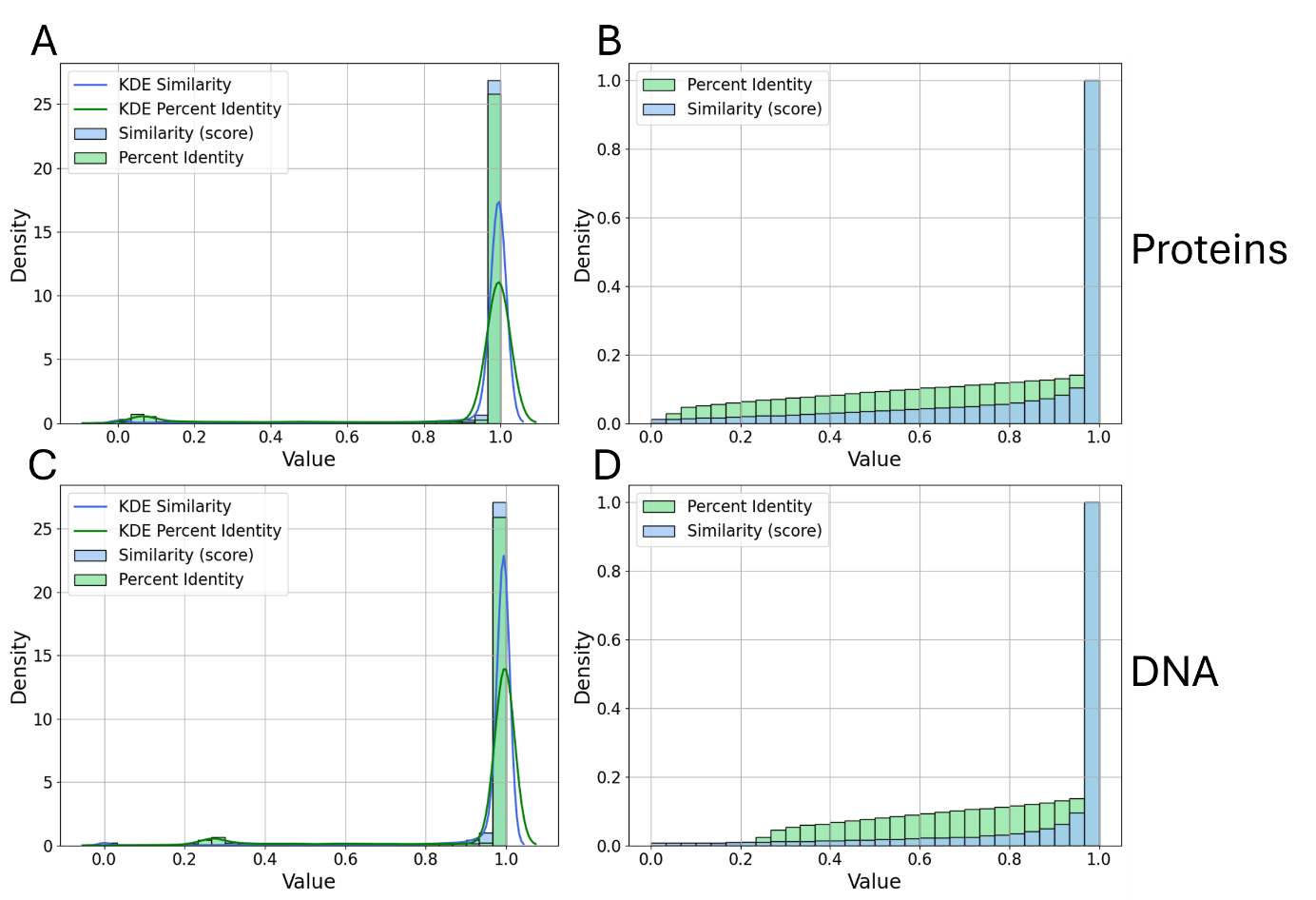


**Fig. S5** Distribution of similarity (SIM) and identity (ID) values (A, C) and their corresponding cumulative distribution curves (B, D) derived from multiple sequence alignments of shared CDS among WUT3, WUT151, SC, and SB. Protein-based analysis is shown in the first row (A, B), while DNA-based analysis is shown in the second row (C, D).
