# Supplementary material for: Genomic insights into the probiotic potential of dairy-associated Saccharomyces cerevisiae WUT3 and WUT151 strains
Source: Funct Integr Genomics. 2026 Jul 6;26(1):177. doi: 10.1007/s10142-026-01963-4 (PMC13333559; doi:10.1007/s10142-026-01963-4)

Functional & Integrative Genomics

**Additional file 2: uncropped gel images**

Genomic Insights into the Probiotic Potential of Dairy-Associated Saccharomyces cerevisiae WUT3 and WUT151 Strains

Aleksander Gryciuk^1^*, Małgorzata Milner-Krawczyk^1^, Adrianna Skoneczna^2^, and Jolanta Mierzejewska^1^**

^1^ Laboratory of Microbiology and Bioengineering, Faculty of Chemistry, Warsaw University of Technology, Warsaw, Poland

^2^ Institute of Biochemistry and Biophysics, Polish Academy of Sciences, Warsaw, Poland

* Correspondence: aleksander.gryciuk.dokt@pw.edu.pl

** Correspondence: jolanta.mierzejewska@pw.edu.pl

1. Raw uncropped electrophoresis gel


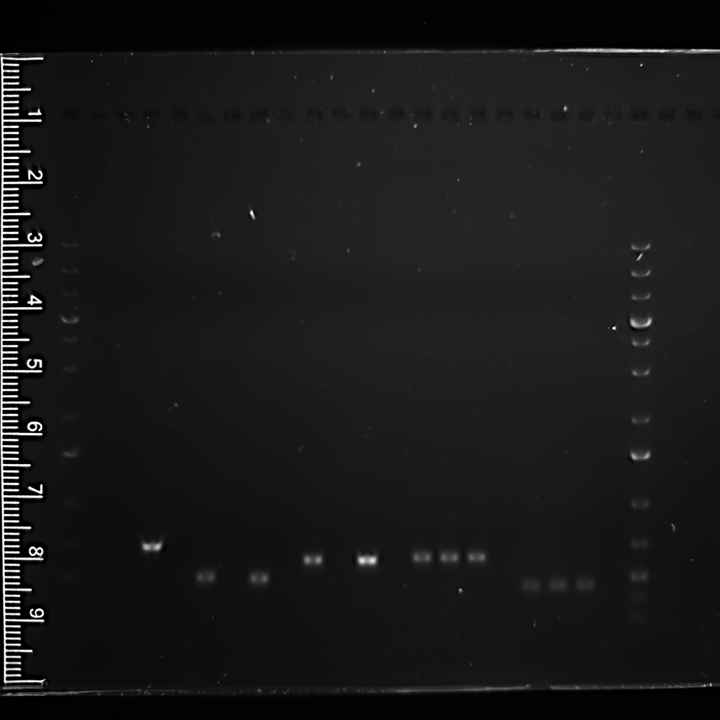


1. Annotated uncropped electrophoresis gel


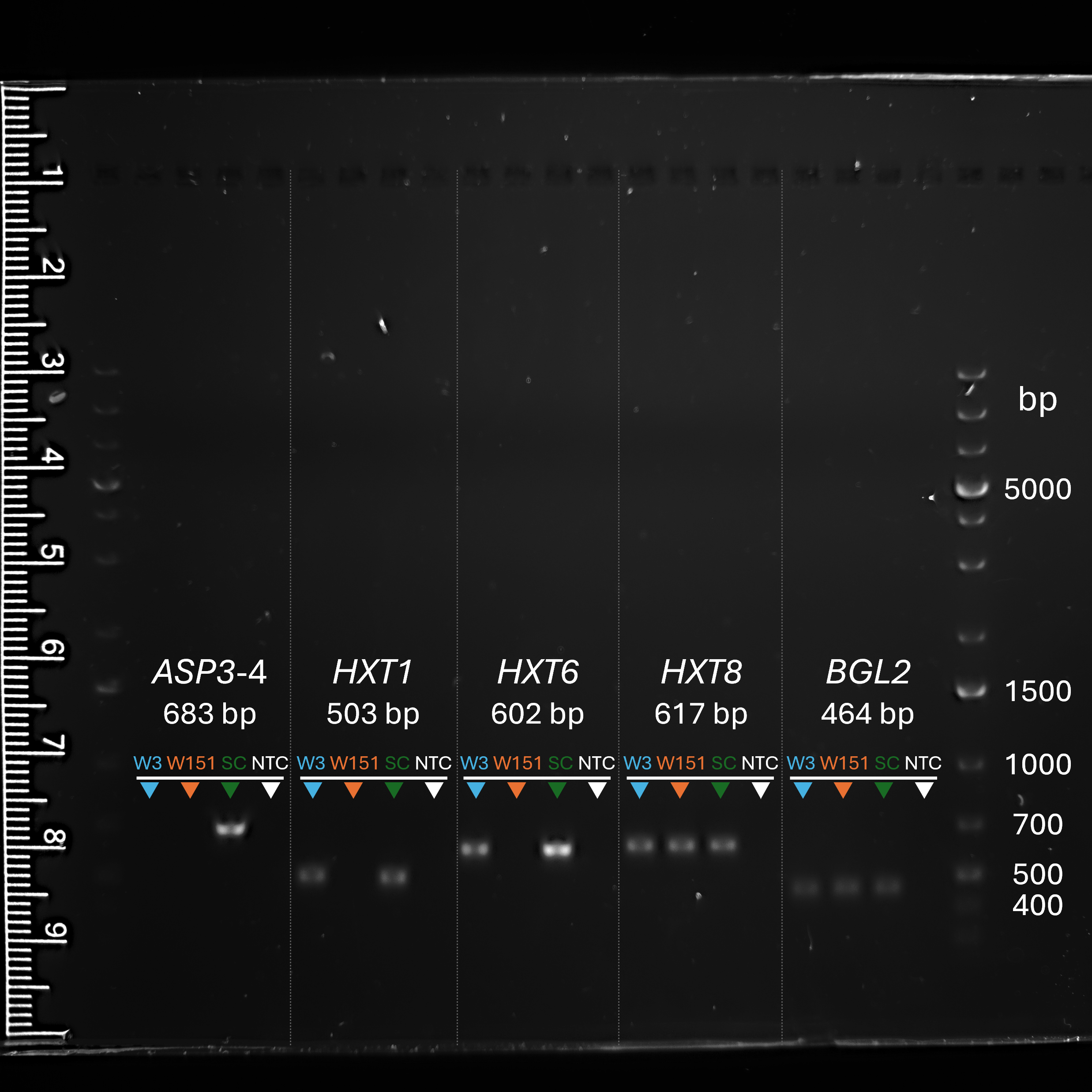

Supplement: Supplementary file 2 — Supplementary Material 2 [file 10142_2026_1963_MOESM2_ESM.docx]
